# Green Synthesis of Metal and Metal Oxide Nanoparticles: A Review of the Principles and Biomedical Applications

**DOI:** 10.3390/ijms242015397

**Published:** 2023-10-20

**Authors:** Denisa-Maria Radulescu, Vasile-Adrian Surdu, Anton Ficai, Denisa Ficai, Alexandru-Mihai Grumezescu, Ecaterina Andronescu

**Affiliations:** 1 Department of Science and Engineering of Oxide Materials and Nanomaterials, Faculty of Chemical Engineering and Biotechnologies, Bucharest National Polytechnic University of Science and Technology, 011061 Bucharest, Romania; denisa.m.radulescu@gmail.com (D.-M.R.); adrian.surdu@upb.ro (V.-A.S.); anton.ficai@upb.ro (A.F.); denisaficai@yahoo.ro (D.F.); grumezescu@yahoo.com (A.-M.G.); 2 Academy of Romanian Scientists, Ilfov 3, 050044 Bucharest, Romania; 3 Research Institute of the University of Bucharest—ICUB, University of Bucharest, 050657 Bucharest, Romania

**Keywords:** green synthesis, metal oxide nanoparticles, biomedical engineering, metal nanoparticles, drug delivery, scaffolds, tissue engineering

## Abstract

In recent years, interest in nanotechnology has increased exponentially due to enhanced progress and technological innovation. In tissue engineering, the development of metallic nanoparticles has been amplified, especially due to their antibacterial properties. Another important characteristic of metal NPs is that they enable high control over the features of the developed scaffolds (optimizing their mechanical strength and offering the controlled release of bioactive agents). Currently, the main concern related to the method of synthesis of metal oxide NPs is the environmental impact. The physical and chemical synthesis uses toxic agents that could generate hazards or exert carcinogenicity/environmental toxicity. Therefore, a greener, cleaner, and more reliable approach is needed. Green synthetic has come as a solution to counter the aforementioned limitations. Nowadays, green synthesis is preferred because it leads to the prevention/minimization of waste, the reduction of derivatives/pollution, and the use of non-toxic (safer) solvents. This method not only uses biomass sources as reducing agents for metal salts. The biomolecules also cover the synthesized NPs or act as in situ capping and reducing agents. Further, their involvement in the formation process reduces toxicity, prevents nanoparticle agglomeration, and improves the antimicrobial activity of the nanomaterial, leading to a possible synergistic effect. This study aims to provide a comprehensive review of the green synthesis of metal and metal oxide nanoparticles, from the synthesis routes, selected solvents, and parameters to their latest application in the biomedical field.

## 1. Introduction

Over the years, nanotechnology has emerged as an essential research field in the biomedical domain. This comes as a result of the latest approaches in bioengineering, which allow the facile manipulation of the developed nanomaterials [1]. Due to their unique properties, such as physiochemical stability, increased surface area, biocompatibility, and non-toxicity, NPs are largely applied as materials in the cosmetics, pharmaceuticals, and tissue engineering industries compared to larger particles [2].

The most studied material groups are metallic and oxide nanoparticles (NPs). Numerous research studies have shown that metals (e.g., Cu, Ag, Pt, Au, Pt, Mg, Zn, etc.) and metal oxides (e.g., ZnO, TiO_2_, CuO, Ag_2_O, etc.) NPs have demonstrated significant therapeutic benefits. These benefits led to their application in various fields, such as drug delivery systems, biosensors, diagnostic imaging applications, and scaffolds for tissue engineering [3,4].

The main metal and metal oxide NP synthesis routes are represented by chemical and physical techniques. In the case of chemical routes, precipitation, sol-gel method, chemical reduction, and polyol synthesis are the most studied, whereas in the case of physical synthesis routes, there was evidence of microwave-assisted combustion, laser evaporation, and pulsed laser deposition [5,6].

However, these methods come with many limitations, such as the use of toxic substances, cost-ineffectiveness, unsafe procedures, and the production of dangerous by-products that are environmentally harmful. The complex nature of the procedures involved in the mentioned synthesis routes and their drawbacks disrupt the biocompatibility of nanoparticles. Therefore, it is urgently necessary to develop a cost-effective, one-step, reliable, biocompatible, and non-toxic method of synthesizing environmentally friendly NPs. One research direction uses biological methods that apply enzymes, plant extracts, and microorganisms [6,7].

The steps taken in green synthesis methods for metallic NPs aim to reduce or exclude the use of toxic substances and solvents by replacing them with eco-friendly metabolites and biomolecules inspired by synthesis routes such as biomimetic or biological synthesis [8].

### 1.1. Overview of the Use of Nanoparticles in Tissue Engineering

In the field of tissue engineering, the development of metallic and metal oxide NPs has been amplified, especially for their antibacterial properties [9,10].

In this direction, NPs have been used to aid many functions in tissue engineering, from enhancing mechanical, electrical, and biological properties to DNA transfection, gene delivery, patterning of cells, and viral transduction. The main purpose of NPs is to facilitate the growth of different tissue types or serve for biosensing or molecular detection. Furthermore, the use of NPs in tissue engineering can also enhance the properties of the scaffolds and provide additional properties, depending on the desired application. The additional properties are given because of their small size and their association with their large surface-to-volume ratio. Thus, the NPs can diffuse easily across membranes and, therefore, facilitate cell uptake. In addition, NPs are also able to mimic the scale of extracellular matrix components in native tissues [11,12,13,14].

Another important characteristic of metal NPs is the high control over the features of the obtained scaffolds, such as optimizing their mechanical strength and offering the possibility of controlled release of bioactive agents [15]. Even though many studies concluded that metal NPs exhibited higher toxicity, recent research has demonstrated that the proper dosage, size, and distribution may reduce the toxicity of the materials. Due to their nature and ease of functionalization, the main features of these materials could be improved to provide a more efficient therapeutic effect [16,17]. The main goal of their optimization was to pursue appropriate mechanical and biological features that make them ideal materials for the targeted application by creating distinct conditions. Therefore, the developed NPs provide adjustable surface properties and a high surface area, increasing the scaffolds’ antiseptic, antibacterial, and mechanical strength to overcome the main barriers of tissue engineering. Until now, many metal/metal oxide NPs have been applied to modify and improve scaffolds’ performance. The scaffolds often combine polymers, ceramic NPs, polymeric NPs, and others [14,15,18].

**Figure 1:**
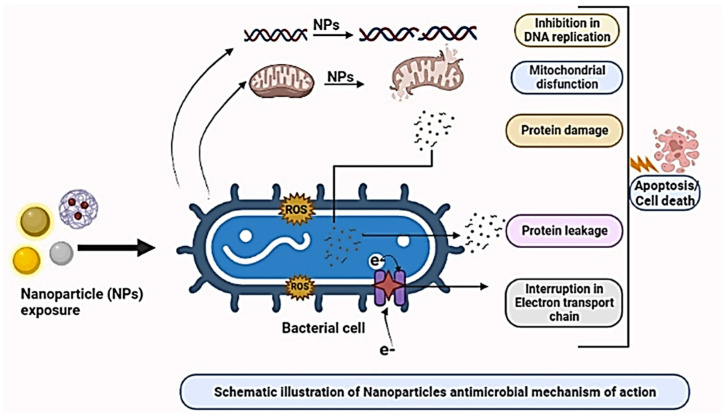
Schematic mechanism: action of nanoparticles on bacterial cells and induction of various cellular mechanisms for apoptosis [20].

Furthermore, nanoparticles can also exhibit antimicrobial properties that, in combination with wound regeneration abilities, make them unique materials that could aid the healing process of wounds. In this direction, green synthesized NPs received increased attention due to their environmental friendliness and great efficacy, making the manufactured material promising wound healing agents [19]. Moreover, Mujahid et al. [20] confirmed the successful use of metal and metal oxide NPs in drug delivery applications due to their antimicrobial, antioxidant, antibacterial, anticancerous, and antiviral activity. Thus, Figure 1 illustrates the antimicrobial activity mechanism of nanoparticles. This mechanism provides a successful route to overcome antibiotic resistance, antibiotic modification, and the inactivation of biofilm formation.

Metallic NPs penetrate the bacterial cell wall and exhibit bactericidal effects. Hence, apoptosis, or cell death, of microbes is generated due to the combined effect of internal biological processes disrupted by NPs. These main processes of the antimicrobial mechanism are represented by the inhibition of DNA replication, protein leakage, mitochondrial dysfunction, protein damage, membrane disruption, or direct entry of NP through the cell wall, and disruption in the electron transport chain (ETC) [21,22,23].

**Figure 2:**
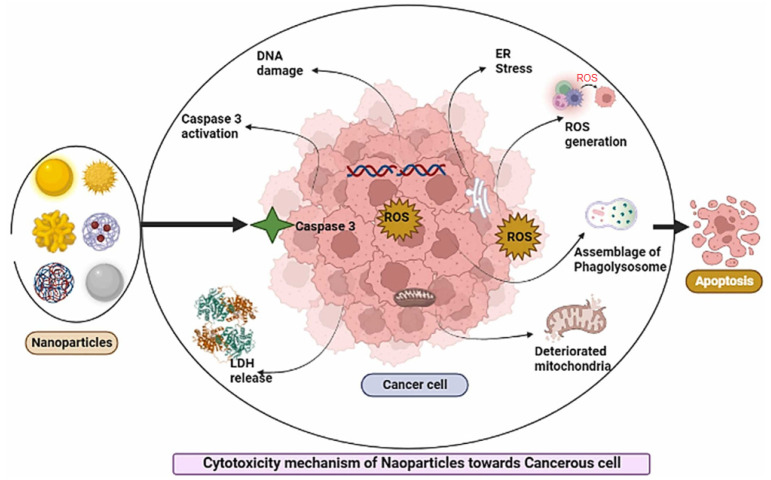
Schematic representation of the anticancer activity of nanoparticles [20].

Related to the anticancer activity of NPs, Mujahid et al. [20] explained the mechanism according to Figure 2. Against cancerous cells, nanoparticles induce the Caspase 3 pathway. This leads to diverse cellular processes and mechanisms, such as reactive oxygen species (ROS) generation, ER (Endoplasmic reticulum) stress, autophagolysosomal accumulation, LDH (Lactate Dehydrogenase) release, and mitochondrial disruption. All these processes lead to the collapse of the cellular framework and, finally, cell death.

### 1.2. The Importance of Environmentally-Friendly Synthesis Methods

Nowadays, the main concern about the synthesis method of metal oxide NPs is the environmental impact. As mentioned before, chemical or physical strategies [24] use toxic agents that could produce potential hazards and exert carcinogenicity or environmental toxicity. These effects could be caused mainly by using hazardous substances such as organic solvents, reducing agents, or stabilizers. Furthermore, the involvement of toxic solvents limits the application of the developed nanomaterials in numerous biomedical or clinical applications. Hence, a more eco-friendly, cleaner, and reliable approach is needed [25,26].

Green synthesis pathways have attracted significant attention in the research field to counter these limitations. Currently, the use of green synthesis is preferred as it leads to the reduction of derivatives/pollution, prevention/minimization of waste, and the use of non-toxic (safer) solvents. Among the available green synthesis methods of metal oxide NPs, using plant extract and plant-derived materials is the most researched technique, with an easy and simple fabrication process that could be reproduced on larger scales [27,28,29]. In addition, the process can be easily scaled up with no unwanted by-products using harmless, universally accepted solvents (e.g., water) and a moderate reaction medium (pH). Another advantage of green synthesis of metal oxide NPs is represented by the necessity of fewer purification steps without any aggressive procedures such as vacuum conditions, high pressure, or high energy [6].

In recent years, there has been a growing interest in developing green synthesis methods due to their numerous advantages over conventional approaches. Furthermore, it is important to note that while there has been significant research on the green synthesis of metal and metal oxide nanoparticles for biomedical applications, the focus has predominantly been on their synthesis methods, physicochemical properties, and potential applications. However, the parameters that influence their synthesis and the biological effects of these nanoparticles have not yet received the same level of attention and investigation. Understanding the biological effects of nanoparticles is crucial for their safe and effective use in biomedical applications. Critical considerations include nanoparticle-cell interactions, biocompatibility, toxicity, and long-term effects on living systems. Despite their immense potential, the biological implications of green-synthesized metal and metal oxide nanoparticles have been relatively understudied. In this study, we aim to bridge this gap by discussing the limited existing research on the biological effects of these nanoparticles. By highlighting the importance of comprehensive biocompatibility studies and toxicity assessments, we hope to draw attention to this crucial aspect that has been overlooked thus far.

## 2. Green Synthesis of Metal Oxide Nanoparticles

### 2.1. Introduction to Green Synthesis Routes for Metal Oxide Nanoparticles

The basics of green synthesis were established in the early 2000s by Anastas and Warner, who presented in their book “Green Chemistry”, the following 12 principles of green chemistry, which are:

1. Prevention—Measures must be taken to prevent the generation of waste;
2. Atom Economy—As much as possible, the materials used for synthesis should be introduced into the final product;
3. Less Hazardous Chemical Synthesis—Priority should be given to synthesis methods that require materials with minimal or no toxicity to the individual or environment;
4. Designing Safer Chemicals—Materials should be designed to reach function with limited or no toxicity;
5. Safer Solvents—The use of auxiliary chemicals or solvents should be avoided when possible;
6. Design for Energy Efficiency—The use of energy should be limited for synthesis;
7. Use of Renewable Feedstocks—a feedstock should be renewable, and depletion should be avoided whenever possible;
8. Reduce Derivatives—Derivatives such as blocking agents and protecting/deprotecting groups should be avoided if possible as they are the cause of additional waste;
9. Catalysis—Catalysis agents are preferable over stoichiometric agents;
10. Design for Degradation—Chemicals must be designed so that, at the end of synthesis, they break down into non-toxic derivatives;
11. Real-time Analysis for Pollution Prevention—The synthesis should be monitored in real-time for the production of toxic chemicals;
12. Inherently Safer Chemistry for Accident Prevention—Agents used in product synthesis should be selected to limit the possibility of dangerous accidents [30].

The principle of environmentally friendly approaches for metal or metal oxide nanoparticle synthesis is the reduction of metal complexes in dilute solutions. The process aims to obtain metal colloidal dispersions. Due to the presence of certain chemicals that can reduce the metal precursor to the desired nanoparticles, natural or green sources such as microbial and plant extracts have received significant interest [31]. Moreover, the green pathway of nanoparticle formation not only uses plant, algae, fungi, or microorganism-based materials as reducing agents, but they also cover the synthesized NPs or even act as in situ capping. Table 1 presents the main biomass sources for synthesizing metal and metal oxide NPs. Their involvement in the formation process reduces toxicity, prevents nanoparticle agglomeration, and improves the antimicrobial activity of the nanomaterial, leading to a possible synergistic effect [32,33].

Another important advance in green synthesis is the ability of biomass sources to hinder NP agglomeration by forming a monolayer around the nanoparticles, an area where surface energy should be maintained. Moreover, the natural compounds from the extract strongly influence the size and distribution of the NPs, enhance the reducing agent present in the extract, and also increase the reaction rate, which helps in the synthesis of nanoparticles with smaller sizes [31].

On the other hand, the green synthesis of nanoparticles also has several drawbacks that are presented in Table 2. In this regard, it was well established that green synthesis is more advantageous than traditional synthesis methods as it is known as a less expensive, environmentally friendly, and harmless approach. Any green synthesis approach operates under mild reaction conditions, such as lower temperatures and pressures. This not only reduces energy consumption but also enhances the overall safety and stability of the process. However, green synthesis may not be suitable for all types of reactions or desired products. Some complex reactions or molecules may require harsher conditions or specific reagents incompatible with green synthesis principles. Green synthesis methods can be more complex than traditional methods, requiring specialized knowledge and expertise. This can pose a barrier to adoption for researchers or industries unfamiliar with green chemistry principles [31].

It is important to note that the advantages and disadvantages of green synthesis may vary depending on the specific method, reaction, and desired outcome. Continuous research and development in the field aim to address these limitations and further enhance the applicability and effectiveness of green synthesis techniques.

#### 2.1.1. Plant-Mediated Green Synthesis

Plant-mediated green synthesis techniques use different plant parts, such as the seed, fruit, callus, bark, stem, flowers, and leaves, to synthesize metal and metal oxide NPs of different sizes and shapes [35].

**Figure 3:**
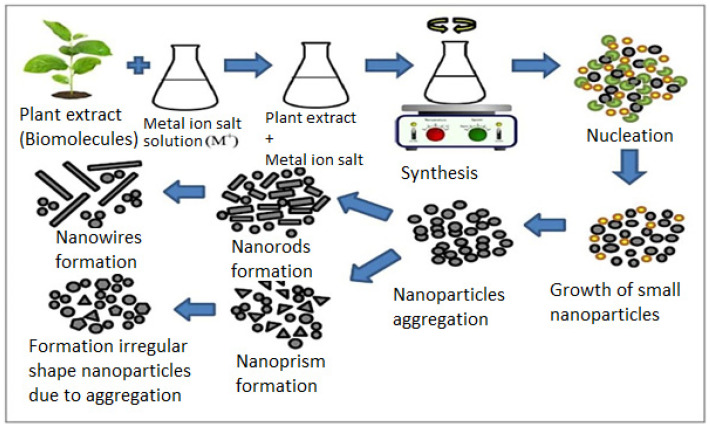
The plant-based synthesis mechanism for metal and metal oxide nanoparticles [37].

Various metabolites incorporated in plant extracts act as stabilizing and reducing agents during metallic NP synthesis. It is well known that bioreduction is a complex process. The biomolecules in the extract perform as a reducing agent by providing electrons to the metal ions, leading to their reduction to the elemental metal. The formed atoms operate as a nucleation center, followed by a growth period in which adjacent smaller particles combine to create larger NPs. In this regard, plant extracts have the ability to stabilize NPs in the final stage of synthesis, ultimately determining their energetically stable and favorable morphology. Further, to inhibit further growth and maintain the particle in the nanoscale range, a capping agent is added. Thus, biomolecules in the plant extract can serve as a reducing agent, or the same molecules can serve as both a reducing agent and a capping agent. Considering all these aspects, the principle of plant-based synthesis has been explained in Figure 3 [36,37].

Additionally, compared with microbial-based synthesis, the nanoparticles fabricated using plant and plant extract have higher stability and a faster reaction time [38].

#### 2.1.2. Bacteria-Mediated Green Synthesis

In another approach to the green route of synthesis of NPs, microorganisms such as bacteria are involved. Nanoparticle synthesis pathways using microbes include combinations of basic cellular biochemistry, metal ion transport (inside and outside cells), microbial resistance mechanisms to toxic metals and activated metal binding sites, accumulation of intracellular metal ions, and metal oxide nucleation [39]. Bacteria are known as efficient organisms due to their ability to produce high amounts of amino acids and enzymes. Moreover, bacteria contain polysaccharides and vitamins that could act as metal ion-reducing agents. In addition to cells, bacteria can chemically detoxify themselves and grow in high concentrations of toxic metals [40]. These microorganisms possess the capacity to produce organic matter inside and transport it outside their cells.

Furthermore, microorganisms have great potential as inexpensive, non-toxic, and environmentally friendly materials that do not demand much energy for synthesizing metal and metal oxide NPs, compared to the physicochemical synthesis techniques. Among the various mechanisms for the green synthesis of nanoparticles, the ones that perform extracellular synthesis are particularly important. This route eliminates the necessity of expensive and complex downstream processing steps to recover intracellular nanoparticles [41].

In recent years, numerous studies have demonstrated the successful synthesis of gold (Au) and silver (Ag) NPs by using bacteria such as *Brevibacterium frigoritolerans*, *Pseudomonas deptenis*, *Bacillus methylotrophicus*, *Visella oriza*, and *Bhargavaea indica*. Nevertheless, the main disadvantage of bacteria-based synthesis includes the difficult steps such as isolation, microbial sampling, storage, and culturing [42].

#### 2.1.3. Algae-Mediated Green Synthesis

Algae-based synthesis is another eco-friendly fabrication method for metal and metal oxide NPs development. Compared to bacteria-based synthesis, algae do not require cellular maintenance. Algae are significant phytochemical sources used in the synthesis of metal nanoparticles. On the other hand, cyanobacteria and microalgae are gaining interest in green synthesis due to their heavy metal hyperaccumulation, considerably higher CO_2_ sequestration rate, lack of toxic byproducts, use of biomolecules (pigments and enzymes) as capping and reducing agents, and also for their low energy input. Since algae are excellent phytonutrients, proteins, pigments, and other nutrient sources, the synthesis process has improved over time, which serves as a significant bio-manufacturer for nanoparticle production [43].

Furthermore, microalgae can generate phytochemicals such as ROS and metal-chelating agents that will interact with nanosized metal nuclei. The interaction between microalgae and metal nuclei occurs at high metal levels [44]. The use of algae as a raw source of synthesis provides many advantages, such as their capacity to grow faster, low cost, ease of use, and non-toxic character, which make them suitable candidates for NPs development. As a natural capping and reducing agent, the algae extract acts as a living cell factory for synthesizing NPs [45,46]. Additionally, due to the secondary metabolites present in algae (plant acids, flavonoids, phenolics, terpenoids, alkaloids, and glycosides) that contain numerous biological effects, the developed materials could be used as nutrition, stimulants, preservatives, antioxidants, insecticides, anticancer, antiviral, and antibacterial agents [43].

#### 2.1.4. Fungi-Mediated Green Synthesis

The fungal-mediated green synthesis of metal/metal oxide NPs is also a highly effective process for monodispersed nanoparticle generation with well-defined morphologies. Fungi act as better biological agents for the preparation of NPs due to the presence of diverse intracellular enzymes. Competent fungi can synthesize larger quantities of NPs compared to bacteria. Furthermore, fungi have many advantages over other organisms due to the presence of proteins/enzymes/reducing components in the structure of the cell. The mechanism of metal nanoparticle formation is enzymatic reduction (reductase) in the cell wall or inside the fungal cell [27]. However, this fabrication method has several setbacks, including synthesis conditions, material selection, product quality control, and application. The previously mentioned parameters are the main challenges for transitioning to large-scale application and industrial production of green-synthesized nanoscale metallic materials. Hence, the main parameters and factors influencing green synthesis will be further discussed in the following section [26].

### 2.2. Solvents Used in Green Synthesis

One of the most vital factors that influence the green synthesis of nanoparticles is represented by the solvents used and their proper selection. In this regard, water has always been a suitable and ideal solvent for all types of synthesis. Between those, ionic liquids, supercritical fluids, and even deep eutectic solvents can be used to synthesize NPs.

#### 2.2.1. Water as a Green Solvent

In the last few years, water has been selected as a universal solvent in NP synthesis. Water has been preferred as a solvent because it is the cheapest, most commonly available, and most accessible solvent on earth. Most ionic compounds dissociate well in water, leading to decreased ion-ion attractive forces and, as a result, causing them to move freely in solution. However, water at various temperatures and a pressure of 10 MPa has more solvency for almost all inorganic compounds than any other solvent. Hence, water can be defined as the most environmentally friendly and safest solvent [47]. Moreover, it is an excellent choice for polar compound solubilization. In this direction, water can even dissolve several organic fragments, such as proteins and sugars, becoming, therefore, the first choice for the aqueous extraction and synthesis reaction [48].

Additionally, water plays a crucial role during crystal structure evolution, particularly during the nucleation and growth phases. The aforementioned phases comprise sub-phases such as self-assembly, elemental distribution in metal particles, coalescence, particle aggregation, and formation pores in NPs [49,50].

#### 2.2.2. Ionic Liquids as a Green Solvent

Given the above context, it can be expressed that there are two major systems used as solvents: water (as the primary green solvent) and bio-based formulation/solvent, which is considered an auxiliary/secondary solvent component. In addition to water, the greener concern of bio-based ionic liquids is based on their renewability and widely available resources such as feedstocks and biorefineries [51].

The main benefits of using ionic liquids as solvents are the following:

1. Many metal catalysts, gases, and polar organic compounds are easily dissolved to support biocatalysts;
2. Ionic liquids have the constructive thermal stability to function over a wide temperature range. Most of them melt below room temperature and begin to decompose above 300 or 400 °C. As a result, they allow a wider synthesis temperature range (e.g., three to four times higher) than that of water.
3. The solubility properties of these solvents can be modulated by changing the anions and cations associated with them.
4. Unlike other alcohols or polar solvents, ionic liquids are uncoordinated, even though they have polarities comparable to alcohol.
5. Ionic liquids do not evaporate into the medium, like volatile solvents, because they have no vapor pressure.
6. Ionic liquids have dual functionality because they contain both anions and cations [27].

In this direction, ionic liquids have emerged as one of the most suitable green mediums for developing NPs. They can provide comprehensive solubility for a wide range of bio-based solvents and, therefore, play exactly as a solvent for the reduction, capping, and stabilization agents of metallic NPs. Furthermore, the equilibrium and reaction rate are also influenced by the dielectric constant, resulting in the narrow NP size distribution achieved by supercritical hydrothermal synthesis. However, these types of solvents are ion-laced with melting points of ~100 °C and are labeled as “room-temperature ionic liquids”. Other studies related that the group of Ali et al. has also confirmed the successful synthesis of Ag, Au, ruthenium (Ru), aluminum (Al), ruthenium (Ru), and platinum (Pt) NPs by using ionic liquids as reducing and protective agents [27,47,52,53].

Another good example of metallic NP synthesis is represented by the study performed by Bussamara et al. [54], where they achieved the successful synthesis of manganese oxide (Mn_3_O_4_) NPs using imidazolium ionic liquids and oleylamine (a conventional solvent). The smaller NPs (9.9  ±  1.8 nm) with improved dispersity have been obtained in imidazolium ionic liquids rather than oleylamine (12.1  ±  3.0 nm).

Furthermore, Lazarus et al. obtained Ag nanoparticles using BmimBF4. The synthesized NPs were observed in large anisotropic hexagonal shapes and smaller isotropic spherical shapes [55]. For the first time, Singh et al. [27] developed a one-step preparation technique for Au and Pt nanoparticles via thiol-functionalized ionic liquids (TFILs). The TFILs acted as stabilizing agents to create crystalline structures with small dimensions. The group also related that, in another study, Dupont et al. [27] selected 1-n-butyl-3-methylimidazolium hexafluorophosphate (room temperature ionic liquid) for the synthesis of iridium NPs by Ir(I) reduction with a small particle size of ~2 nm. All these studies presented the effectiveness of ionic liquids to be applied in green synthesis routes.

#### 2.2.3. Supercritical Fluids as Green Solvent

Supercritical fluid (SCF) technology contributes significantly to the increased sustainability of a wide spectrum of synthesis and separation processes while maintaining environmental aspects such as (1) full solvent recycling and (2) no use of organic solvent. Over the years, many studies have concluded that SCFs are initially novel gas-liquid hybrids exhibiting unique characteristics of a green environment for chemical reactions and separations [47].

In the supercritical state, solvent characteristics (thermal conductivity, density, and viscosity) are considerately altered. CO_2_ is the most feasible supercritical, non-hazardous, and inert fluid [27]. Additionally, supercritical fluids can dissolve various solutes as liquids and still possess gaseous properties such as high diffusivity and zero surface tension. Remarkably, SCFs can be mixed with gases, while the solubility can be programmed by adjusting the pressure and temperature. Beyond these, the aforementioned properties suggest that SCFs are attractive media for the targeted delivery of reactant molecules to target areas, such as intricate surfaces, areas with high aspect ratios, and poorly wettable substrates, to achieve remarkable uniformity and homogeneity. To confirm the applicability of SCFs, the research group led by Ali has also presented Sc-CO_2_ as a green medium for separation and chemical reactions. Hence, CO_2_ is one of the most feasible non-hazardous inert and supercritical fluids used for the synthesis of copper and silver NPs in their supercritical states, suggesting, therefore, a solubility decrease of metal oxides ~ the critical point, further leading to the supersaturation, formation, and configuration of the developed NPs. Further, Kim et al. have also presented the synthesis process of crystalline tungsten oxide (WO_3_) and tungsten blue oxide nanoparticles using an Sc-W system. In addition, they have confirmed that Sc methanol was a more suitable candidate compared to the hydrothermal reduction of glycerol used to reduce WO_3_. Thus, inspired by the comprehensive merits of Sc-W and Sc-CO_2_ solutions, many SCF-based approaches have been developed for nanomaterial synthesis [27,47]. As another example, supercritical water can also serve as a good solvent system for many reactions, as it has a critical temperature of 646 K and a pressure of 22.1 MPa [56].

#### 2.2.4. Deep Eutectic Solvents

Deep eutectic solvents (DES) have been classified as an alternative category of ionic fluids that embrace the principles of green chemistry. In this regard, they are typically more synthetically accessible, less expensive (typically from bulk base chemicals using solvent-free processes/waste), biodegradable, and non-toxic [57]. DESs can be obtained from environmentally renewable raw materials such as chloride, choline, carbohydrates, or amino acids. These types of solvents contain at least two organic molecular components, namely Lewis/Bronsted base and acid, which act as hydrogen bond donors and acceptors, respectively [58].

In general, it can be debated that DESs represent a sustainable class of emerging solvents and are therefore considered green solvents, such as glymes, glycerol, polyethylene glycol, supercritical fluids, and room-temperature ionic liquids (RTILs). Yet, DES are not completely RTILs. They are composed of molecular components instead of predominantly ions. The DES’s charged components are designed to tune the nucleation and growth mechanisms by neutralizing charge, altering reduction potentials, and dictating growth along programmed/desired crystallographic directions [59]. In addition, the biocompatibility of DESs has guaranteed the fascinating potential of designing biomolecular architectures in these new environments. DES plays an important role as designer solvents while developing pre-designed/desired nanoscale structures such as size-controlled and defined-morphology NPs, colloidal and porous assemblies, metal-organic nanocomposites, and NP-based DNA/RNA templates. Further, DESs can direct nanosynthesis by acting as a metal/carbon source, supramolecular template, sacrificial agent (e.g., releasing ammonia from urea), and/or redox agent. All these processes can occur in the absence of a formal stabilizing ligand [60].

Wei et al. provided a tactful and efficient electrochemical deposition route for Pt nanoflowers (200 nm) with pointed crystal petals in DES, namely reline (1:2 choline chloride/urea) at 80 °C. Similarly, the same group has successfully synthesized shape-controlled concave tetrahexahedral Pt NPs in DESs [61]. Subsequently, Hammons et al. [62] used ultra-small-angle X-ray scattering that sustained the electrochemical deposition and stabilization of Pd NPs in the same reline medium. It was observed that the NPs formed by the electrodeposition approach in relining comprise the assembly of nanocrystals into 2D superstructures due to the simultaneous layer-by-layer deposition of a net positive charge over the anionic layer.

Hence, to sum up, Table 3 presents the main advantages and drawbacks of each explained solution.

### 2.3. Factors That Impact Green Synthesis

With time, many researchers have concluded that the process of green synthesis is influenced by many parameters such as pH, temperature, metal ion concentration, reducing agents’ concentration, and the synthesis mechanism (intracellular/extracellular). In addition, pressure, interaction time, and the growth medium type of microorganism also play a vital role in the synthesis of nanoparticles, especially in defining the size, texture, shape, and number of NPs [63].

The pH value of a solution is a critical parameter to be considered in the green synthesis of metal and metal oxide NPs [64]. Further, the pH influences the shape, size, and synthesis rate of the developed nanoparticles [63,64]. Moreover, the nucleation center formation in the green synthesis of NPs is highly dependent on pH. Hence, an increase in pH could lead to an enhancement in the nucleation center, which will thus increase the metal ion reduction to metal nanoparticles (NPs). Further, the reduction time of the metal salt was connected to the pH of the reaction medium, as pH influences the interaction between the metal ion and the functional groups in the plant extract. Furthermore, researchers have shown that the synthesis of smaller-size NPs occurs more in a basic environment than in an acidic solution [64].

A good example of pH influence on the green synthesis of metallic NPs is confirmed through the study performed by Qian et al. [65], where they synthesized Ag NPs using *Penicillium oxalicum* and different pH ranges for comparison. They have concluded that alkaline pH produces NPs with a shorter synthesis time and a smaller size distribution. These characteristics indicate increased stability due to the electrostatic repulsion of the anions. Priyadarshini et al. have also found that the successful synthesis of Au NPs using *Aspergillus terreus* is controlled by pH. At pH 8, the shape of nanoparticles transforms from spherical to rod, with a mean size of 20–29 nm. By increasing the pH to 10, the mean size of NPs with spherical shapes has decreased to 10–19 nm. Hence, the continuous increase in pH triggers an enhanced reduction reaction [66]. Further, Hussain et al. have concluded that pH 7 can be considered an ideal pH value for completely reducing metal salt to metal ions to obtain further materials used for biomedical applications. Furthermore, biosynthesis can occur at pHs ranging from 2 to 14. Related to temperature as a reaction parameter, a room temperature of 25 °C has been demonstrated to be ideal for synthesizing small-sized NPs [67].

Therefore, another factor that could influence the green synthesis of metal and metal oxide NPs is temperature. This parameter can also influence the shape, size, and rate of synthesis and consequently affect nucleation. Further, temperature plays an important role in green nanoparticle synthesis, affecting the morphological properties in the same way as pH. Moreover, temperature also influences nucleation center formation, as mentioned before. In this regard, decreasing the temperature and the formation of nucleation centers also decreases, which in turn causes a decrease in the synthesis rate [68]. Due to the nature of the secondary metabolites existent in the plant extract, room temperature was considered the necessary temperature for nanoparticle synthesis to prevent degradation and distortion of functional groups [69].

However, researchers have demonstrated that triangular-shaped NPs are produced at lower temperatures, while spherical-shaped NPs are produced at higher temperatures. Akintelu et al. [64] established that a smaller volume of plant extract is required to synthesize stable NPs at higher temperatures and that larger-sized NPs are obtained at higher temperatures. For example, other studies related that Bala et al. observed that changing the heat treatment temperature resulted in different sizes and morphologies of ZnO NPs. Heat treatment at 30 °C proved irregular morphology and low crystallinity of the particles. Yet, the NPs synthesized with heat treatment at 60 °C and 100 °C showed high crystallinity and agglomerated nanoparticles in cauliflower-like and dumbbell-like morphologies, respectively. These variations are most likely related to the fact that higher temperatures increase the nucleation rate of crystal formation. In accordance, the same group explained that Parra and Haque synthesized ZnO NPs through chemical routes. They have noticed that the higher the temperature, the faster the crystal growth and nucleation rate are, causing nanoparticle agglomeration and larger particle sizes. Another characteristic related to agglomeration is that the time interval of the heat treatment can affect the formation of clusters. Similarly, they have also explained that Dhadapani et al. demonstrated that by increasing the heat treatment time performed at 50 °C from 30 to 90 min, the growth of agglomerates and particles also increased. These results confirm the results observed in different chemical synthesis methods, where increasing the nucleation time resulted in the formation of larger ZnO particles [70].

Furthermore, another study conducted by Rana et al. related that the group led by Sheny proved that NPs synthesized from *Anacardium occidentale* leaf extract are influenced by temperature. At 27 °C, 2.5 mL of plant extract was necessary, while at 100 °C, only 0.6 mL of extract was found to be sufficient for their synthesis. Additionally, they have also mentioned that Riddin et al. concluded that a lower amount of Pt NPs was synthesized by fungi at 65 °C compared to 35 °C. The same group also explained that in another experiment, Von White et al. synthesized Au NPs, where the synthesis process was preferred at a lower temperature (25 °C). Furthermore, they have also explained that Singh et al. [63] reported that the stability of Ag NPs derived from a strain of *Brevibacterium frigoritolerans DC2* was found to be maximum at room temperature and in the pH range from 4 to 10.

Subsequently, the period of reaction and incubation time significantly impact the morphological properties, quality, and yield of the synthesized NPs. Changes in incubation time, in combination with storage conditions, also influence the properties of the synthesized NPs. Many studies suggest a prolonged incubation period might induce aggregation and reduction potential in the synthesis process. The concentration of plant extracts is another factor vital in synthesizing metallic NPs via green synthesis methods. The plant extract acts as an electron source for reducing the metal ion. The decrease in the volume of the plant extract triggers the lower formation of NPs. Moreover, using a larger volume of plant extracts in the synthesis process might produce a larger amount of phytochemicals, which are necessary for the fast reduction of the metallic salt. However, as the metal salt reduction is increased, the decrease in NP size is enhanced [64,71]. Pressure can also be considered a crucial parameter in the biosynthesis of NPs. The rate of NP synthesis was found to be higher at ambient pressure, indicating that pressure also impacts nanoparticle synthesis. Furthermore, the pressure used for the reaction mixture can also influence the nanoparticle’s morphology [63].

Considering all the aforementioned parameters and routes of preparation for the green synthesis methods, great attention must be paid when trying further to develop nanoparticles for tissue engineering or drug delivery applications. The most studied green synthesized nanoparticles until this point will be further discussed in the following section.

## 3. Green Synthetized Noble Metal Nanoparticles

### 3.1. Gold Nanoparticles: Main Properties and Advantages

Au NPs have drawn increased interest from scholars due to their unique properties, such as thermal, electronic, optic, biological, and chemical features [72]. Over the years, Au NPs have been successfully applied as sensors, catalysis, anticancer, antimicrobial, gene expression, cosmetics, diagnostics, disease diagnosis, therapy, pathogen detection, biolabeling, drug delivery, targeted therapies, and microscopic imaging [73].

Therefore, among all the metal NPs investigated, Au nanoparticles are the most promising for biomedical applications. Their surface atoms allow the incorporation of different targeting moieties such as proteins, peptides, other biomolecules, and drug molecules. Furthermore, the size of nanoparticles can be engineered to permit efficient penetration into specific cell types, including cancer cells, enabling the interrogation, diagnosis, and treatment of diseases at the cellular level. Another advantage of using Au NPs is represented by their properties of being non-toxic and non-immunogenic. The magnetic, radioactive, and photophysical properties of these NPs enable the development of a multitude of therapeutic, diagnostic, and theragnostic agents to treat various diseases [74].

**Figure 4:**
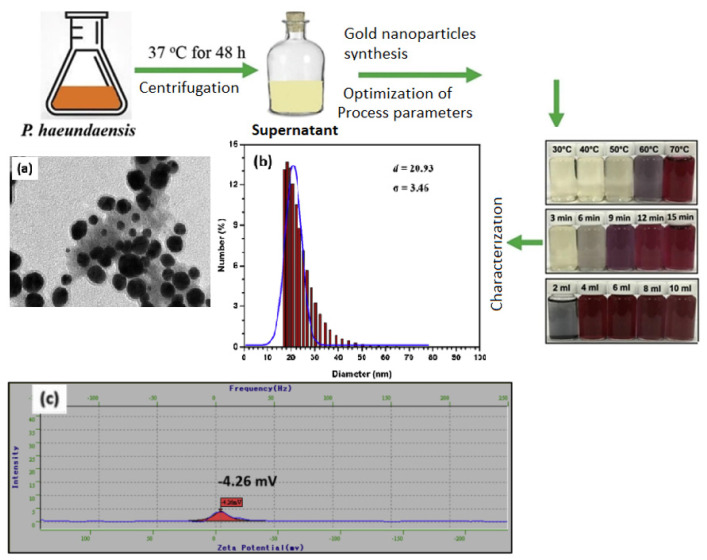
The synthesis process of Au NPs using *Paracoccus haeundaensis BC74171T* [73]. (**a**) TEM image 50 nm scale bar, (**b**) particle size distribution histogram and (**c**) Zeta potential measurement.

Related to their green synthesis, numerous studies have been performed using diverse natural sources, from bacteria to plants or plant extracts. A good example is the research performed by Rodríguez-León et al. [75], where they obtained Au NPS using *Mimosa tenuiflora* extracts. They have concluded that the polyphenolic compounds provide the necessary electrons for Au^3+^ ion reduction to Au^0^ NPs, with sizes ranging from 20 to 200 nm. Another study performed by Shahid et al. [73] related to the successful synthesis of Au NPs using marine bacteria, i.e., *Paracoccus haeundaensis BC74171T*, as shown in Figure 4. Hence, marine microorganisms can adapt to harsh environments and can, therefore, be used in the synthesis process. *Paracoccus haeundaensis* bacteria generated NPs with sizes between 3.46 and 20.93 nm, as confirmed by DLS and TEM characterizations.

As an antifungal material, Kumar et al. [76] have also synthesized Au NPs using *C. caudatus Geisel* extract and chloroauric acid (HAuCl_4_), where the manufactured NPs have provided excellent antifungal activity. Elemike et al. have also demonstrated the anticarcinogenic activity of Au NPs, which have been obtained using *S. ovatum* leaf extract. The size of the NPs was 24 nm, and the effect of Au NPs was evaluated against HeLa cells, where the NPs exerted cytotoxic effects on the growth of carcinoma cells with an IC50 value of 0.0027 μM, while the standard drug (5-fluorouracil) showed an IC50 value of 40 μM. These results demonstrated that green synthesized Au NPs have a higher efficacy in inhibiting the growth of carcinoma cells further. Moreover, green synthesis of Au NPs using *S. ovatum* leaf proved to be a potential alternative method for nanomedicine development as an anti-cancer drug [77,78].

### 3.2. Silver Nanoparticles: Main Properties and Advantages

Another category of metal nanoparticles widely studied in both green synthesis and their application in biomedical fields is represented by Ag NPs. In this direction, Ag NPs are the most commonly used metallic nanoparticles for biomedicine, agriculture, healthcare, environmental applications, and textile coatings. They have exhibited the most prominent antifungal, antioxidant, and antibacterial properties among many categories of nanoparticles. Related to their use in tissue engineering and wound healing applications, Ag NPs prevent wound spreading. The NPs are preferred to be used in ointments to treat damage and burns [79,80]. Due to their capacity to be obtained with different shapes, sizes, and increased surface area, Ag NPs are demonstrated to be a potential candidate for multifaceted tissue engineering and drug delivery applications [81].

Related to the synthesis route, compared to chemical synthesis, the green synthesis pathway has proven to be the most researched domain by using biological entities such as fungal strains, plant extracts, and microbial enzymes. In this regard, Ashique et al. [82] explained that Ag NPs had been successfully synthesized by using plants or plant extracts from *Vitex negunda*, *Brassica rappa*, and *Coccinia indica*. These green plant biomasses can be obtained from plants like *Azadirachta indica*, *Alternanthera dentate*, *Ocimum sanctum*, *Melia dubia*, Mulberry leaves, *Brassica rappa*, *Coccinia indica*, and *Vitex negunda* for tailoring Ag-NPs. These biogenic NPs are considered more stable and reliable for safety issues (compared with bottom-up methods), therefore reducing toxicity and environmental concerns [82,83].

Furthermore, natural phytochemical molecules such as flavonoids, terpenes, phenol ketones, alkaloids, aldehydes, carboxylic acids, and amides are successful antioxidants to be used as sources of reducing and stabilizing agents for Ag NP synthesis [84]. Hence, Dias et al. confirmed that flavonoids can successfully inhibit inflammatory biological enzyme release and, thus, enhance the wound healing process and produce a pain relief effect. Moreover, they can improve blood circulation and reduce blood lipids and sugars [85]. Related to the anticarcinogenic activity of green synthesized Ag NPs, Abbasi et al. have also related the successful formation of the material by using anthocyanin extract derived from purple basil. The developed NPs presented an average size of ~40 nm and increased anticarcinogenic activity against HepG2 liver carcinoma cells [86].

### 3.3. Platinum Nanoparticles: Main Properties and Advantages

**Figure 5:**
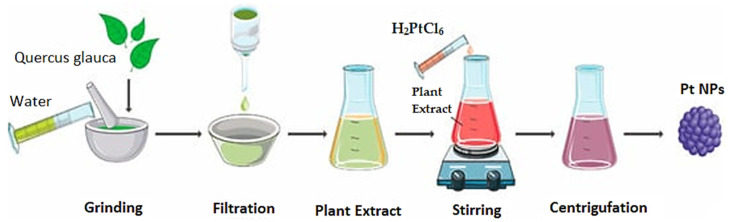
Schematic representation of platinum NPs synthesized using plant extracts [90].

Among many metal NPs, Pt is another noble metal widely researched in various chemical and biological domains due to its properties, such as excellent resistance to corrosion, high surface area, and chemical attack. The currently available information about the antitumor effects, antibacterial properties of Pt NPs, and other potentially beneficial characteristics makes them an interesting subject for comprehensive research [87]. Therefore, Babuska et al. reviewed the applicability of Pt NPs in the biomedical field and their potential applications. Numerous studies concluded that these NPs have unique properties such as biodegradability, biocompatibility, high stability, and osteoconductivity. Furthermore, Pt NPs are known to be used in many other biomedical applications. They can be applied for the detection of cancer cells, the reduction of cellular oxidative stress, and the treatment of Parkinson’s disease. Additionally, Pt NPs have bacteriostatic and cytotoxic effects on cancer cells [88]. Combined with low concentrations of anticancer drugs, green synthesized Pt NPs may provide synergistic anticancer activity or tumor reduction, but novel strategies still need to be investigated for this topic. More studies have reported that nanoparticle production can be indicated by color change. Platinum salts such as K_2_PtCl_6_, H_2_PtCl_6_, K_2_PtCl_4_, PtCl_2_, Pt(NH_3_)_4_-(OH)_2_, Pt(AcAc)_2_, Pt(NH_3_)_4_CI_2_, and Pt(NH_3_)_4_(NO_3_)_2_ are applied for biosynthesis. The biochemical reduction of Pt salts to Pt^0^ relies on the redox reactions carried out by naturally reducing biopolymers. Additionally, these compounds can act not only as reducing agents but also as capping agents or colloidal biostabilizers. The reduction process is performed by many biopolymers: polysaccharides and proteins, as well as aldehydes, alcohols, acids, ketones, biologically active substances, and other metabolic products [89]. Jeyaraj et al. demonstrated the successful synthesis of Pt NPs by using biomass sources, as explained in Figure 5 [90].

Additionally, antibacterial activity can be influenced by the size, morphology, and shape of the nanoparticles, as well as their surface charges. Most metal NPs, such as Au, Ag, Pt, ZnO, Pd, and Cu, have a negative zeta potential and therefore have possible cell-damaging properties. However, even if Pt NPs have a more negative zeta potential and cause severe damage to the cell, they exhibit enhanced antibacterial activity. Hence, the group headed by Jeyaraj has also reported that apigenin-functionalized Pt NPs displayed important antibacterial activity against *Staphylococcus aureus* and *Pseudomonas aeruginosa* [90]. In addition, Table 4 also presents other studies about the green synthesis of noble metal NPs and their biological effects.

## 4. Green Synthetized Metal/Metal Oxide Nanoparticles

### 4.1. Copper Oxide Nanoparticles: Main Properties and Advantages

In the tissue engineering domain, copper (Cu) proved to possess many biological properties by stimulating endothelial cell proliferation during wound healing, promoting the differentiation of mesenchymal stem cells (MSCs) into the osteogenic lineage, regulating vascular endothelial growth factor (VEGF) gene expression, and avoiding infections through its antibacterial properties. Moreover, Cu exhibits specific physicochemical properties, such as improving mechanical strength, porosity, and cross-linking. Therefore, Cu is a promising metal that can be further applied to endow functional properties for cartilage and bone repair. This regeneration could occur by directing cellular behaviors as well as adapting the physicochemical properties of biomaterials [95].

**Figure 6:**
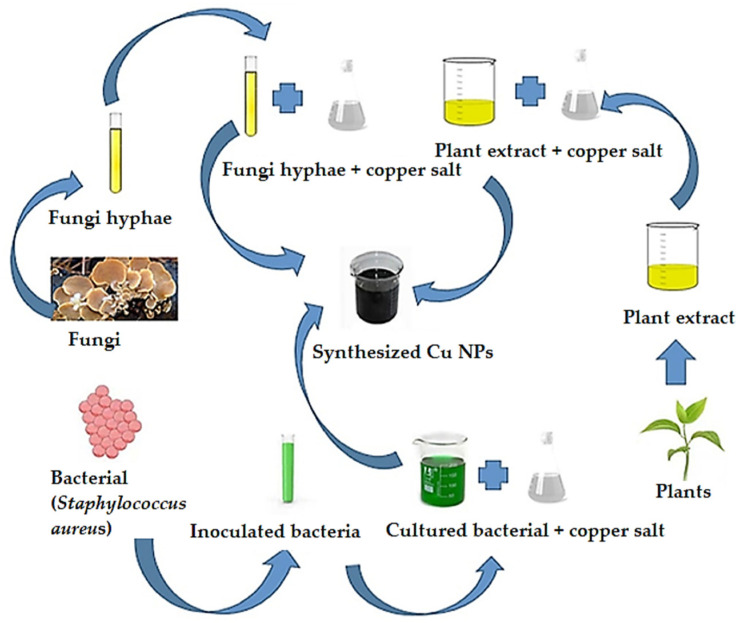
Main green synthesis route of CuO NPs [64].

In recent years, CuO NPs have received significant attention due to various applications, as they can exhibit various biological properties such as antifungal, antiviral, antibacterial, anticancer, and antioxidant activity. For the development of CuO NPs, many physical or chemical synthesis routes have been performed. However, these could cause several side effects, such as the release of highly toxic chemicals into the environment, high costs, and high energy consumption. The need for a more ecological synthesis route led to the continuous research of these nanoparticles to further allow the use of biomass as the primary source of synthesis. Through this ecological approach, various bacteria, plants or plant extracts, fungi, algae, and other biological entities such as alginate, starch, gelatin, ovalbumin, and oleic acid have been used to fabricate CuO NPs, as shown in Figure 6 [96].

The synthesis of CuO NPs using plant extracts has been discussed in detail in numerous studies. Lately, Veisi et al. investigated the CuO NPs synthesis using *Stachys lavandulifolia* flower extract (herbal tea). The synthesized CuO NPs have a mean particle size of 15 nm to 25 nm and a spherical morphology. The phytochemicals found in *Stachys Lavandulifolia* act as bio-reductants of Cu^2+^ ions and stabilizers of Cu^0^ NP [73,97]. Additionally, Sukumar et al. demonstrated the effective development of CuO NPs using *Caesalpinia bonducella* seed extract. The obtained CuO NPs have been further assessed for antibacterial effect with *Aeromonas* (Gram-negative) and *S. aureus* (Gram-positive) bacteria, and in both cases, higher antibacterial activity was observed [98].

Additionally, Cao et al. have obtained composite ZnO-CuO NPs using *S. nigra* and further determined the anticancer properties of the material. The results showed dose-dependent anticancer ability against A375 cells while indicating low toxicity against A549 cells. It was concluded that the lower toxicity could be attributed to the different serum levels of trace elements in each cancer cell [99].

### 4.2. Zinc Oxide Nanoparticles: Main Properties and Advantages

Another highly studied metal oxide is represented by zinc oxide (ZnO). ZnO NPs have been intensively researched in nanomaterials due to their remarkable chemical stability, energy-high exciton binding, wide bandgap, non-toxicity, and biocompatibility [100,101,102]. In addition, zinc is an essential part of the enzymatic activities during nucleic acid and protein synthesis, being therefore non-toxic to healthy cells but active enough to act against cancer cells for apoptosis sensitization [103]. Hence, ZnO NPs, in tissue engineering, act as antibacterial agents that induce gram-negative and gram-positive bacterial cell death through reactive free radical generation and membrane disruption. Further, zinc oxide NPs exhibit excellent antioxidant activity in normal mammalian cells by scavenging reactive free radicals and upregulating antioxidant enzyme activities [101]. In this direction, ZnO NPs can be applied in biomedical applications such as tissue engineering, drug delivery, anticancer, antibacterial, antioxidant, and hypoglycemic agents [104,105].

Related to the synthesis part of these nanoparticles, various physical, chemical, and biological routes are used to develop zinc oxide NPs. According to the literature, the chemical pathways include electro-deposition, wet chemical, chemical micro-emulsion, microwave-assisted combustion, spray pyrolysis, and chemical and direct precipitation. On the other hand, physical methods imply high vacuum usage in processes such as pulsed laser deposition, thermal evaporation, and molecular beam epitaxy [106]. Alternatively, the green synthesis of ZnO NPs is cost-effective, environmentally friendly, easy, upscaleable, and biocompatible. Biological sources such as fungi, plants, algae, etc., have been used to synthesize and act as a capping agent for NPs, essential for their biocompatibility and stability [107].

**Figure 7:**
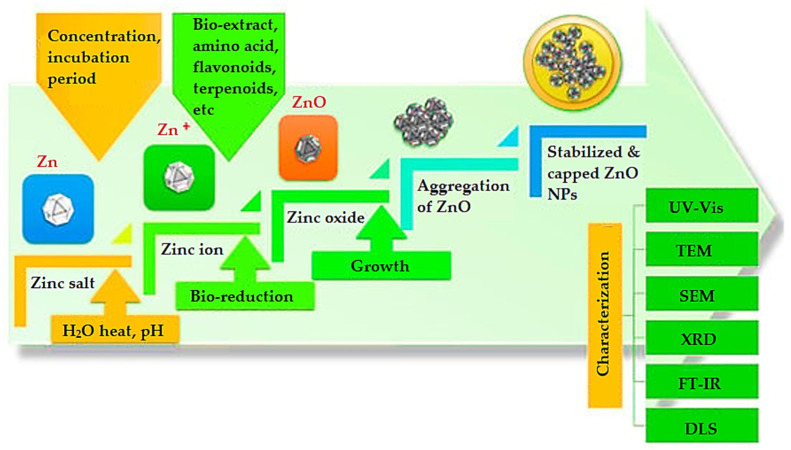
Schematic representation of green synthesis mechanisms of ZnO NPs [109].

Green synthesized NPs exhibit improved anti-bacterial activity compared to the traditionally obtained NPs thanks to the coating of organic compounds such as polysaccharides, quinones, amides, ketone, and aldehydes, which themselves are known to have a substantial antibacterial effect. The synthesis mechanism can be observed in Figure 7. Thus, Agarwal et al. have explained the development of ZnO NPs using lemon. The developed nanoparticles exhibited higher inhibition zones against gram-negative and gram-positive bacteria compared to the chemically synthesized NPs. The coating of phytochemicals on the surface of the synthesized green NPs makes them biologically neutral and compatible. Moreover, using plants and plant extracts to synthesize NPs has been shown to exclude the need to maintain aseptic conditions necessary for the growth of bacterial and fungal cultures [108].

In addition to plant-based primary sources, microorganisms (fungi or algae) or enzymes can also be used to develop biosynthesized NPs. Muthuvel et al. [110] studied ZnO NP synthesis using *Solanum nigrum* leaf extract and water as solvents. They have concluded that the average particle size of green ZnO NPs is smaller than the ones obtained via chemical procedures and also displayed a lower band-gap energy (3.26 eV) as compared with the chemically synthesized Zn ONPs (3.45 eV) [111]. Likewise, the same group has also related that Mazumder et al. developed ZnO NPS (spherical, 11 nm) using the alpha-amylase enzyme in 2 h at 25 °C. They have found that zinc acetate interacts with alpha-amylase and amino acids such as tyrosine (155, 238), glutamine (158), and lysine (209) present in the binding site and performs as a reducing agent to reduce zinc acetate into ZnO NPs [101,112].

### 4.3. Magnesium Oxide Nanoparticles: Main Properties and Advantages

Nowadays, among diverse metal oxide NPs, magnesium oxide nanoparticles (MgO NPs) have gained increased attention due to their exceptional nontoxicity, biocompatibility, high stability under harsh conditions, and remarkable applications, especially in the biomedical field. Furthermore, MgO NPs also provide several valuable physicochemical characteristics, such as a large surface area, high ionic character, oxygen vacancies, and unusual crystal morphology that allow them to interact with numerous biological systems easily. In addition, in biomedicine, MgO has been used for stomach pain treatment, bone regeneration, heartburn relief, and other therapeutic applications such as drugs, coated capsules, biolabeling, blood collection vessels, and many others. Likewise, MgO NPs have been widely researched as they provide unique properties such as antifungal, antibacterial, anticancer, and antioxidant activity. Therefore, there is an urgent need to explore new synthetic strategies for MgO NPs production with less hazardous or toxic side effects [113].

In addition to the properties of MgO NPs, they exhibit increased biocompatibility, biodegradability, high stability, cationic capacity, and redox properties. Therefore, they have become an alluring material to combat microbes and overcome the challenges associated with microbial biofilm removal and antibiotic resistance [114,115]. The antibacterial efficacy of MgO is achieved through various mechanisms that include the direct interaction with the bacterial cell wall, the generation of ROS, and the initiation of intracellular defects such as macromolecular interactions (proteins and DNA) [116].

**Figure 8:**
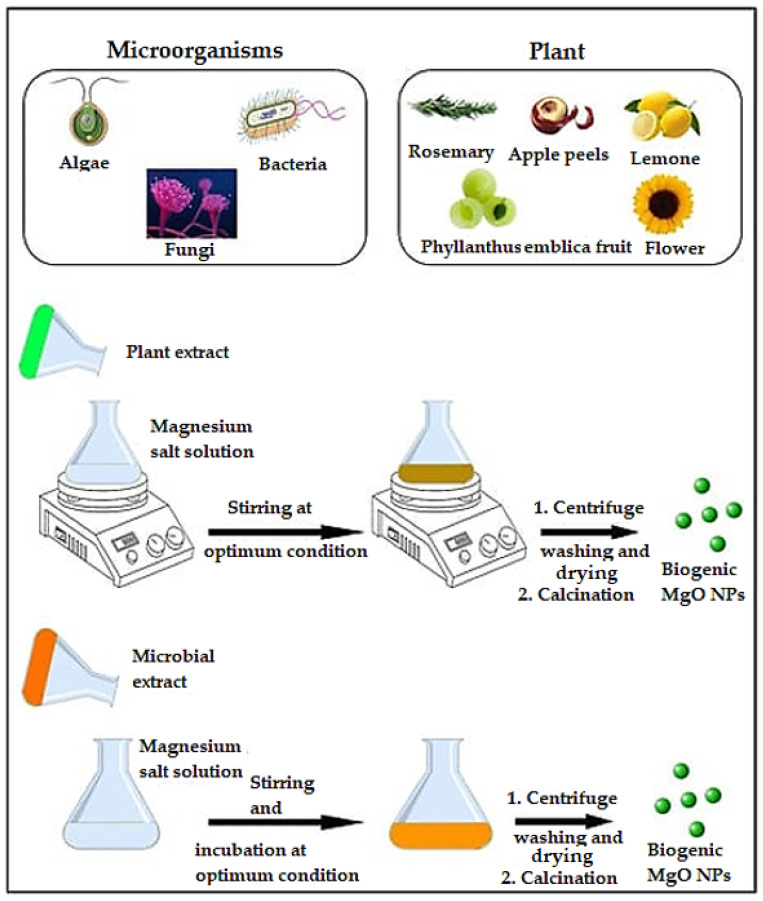
Schematic representation of MgO NPs green synthesis [117].

Related to their synthesis, MgO NPs can be obtained through many physical and chemical approaches. However, in the last couple of years, the greener synthesis, compared to the other methods, has been preferred as the NPs have more stable, environmentally friendly, non-toxic, and cost-effective properties. The main biosources and synthesis mechanisms have been explained in Figure 8 [114,117].

In this regard, Kumar et al. demonstrated the use of *Camellia sinensis* (tea leaves) extract as a reducing agent for synthesizing MgO NPs. In this case, the extract from tea leaves provides the necessary polyphenols that aid the metal salt precursor reduction. The manufactured NPs exhibited a spherical morphology with an average particle size of 65 ± 5 nm [118].

Additionally, Kumar et al. mentioned how Taghavi and his group conducted a study about the development of MgO NPs using Acacia (Arabic) gum. Gum Arabic is used to reduce the precursor for MgO NP extraction, reducing the size of magnesium oxide nanoparticles to ~5.3 nm. The same group has also mentioned how another example of the successful development of green synthesized MgO NPs is explained in the study performed by Dobrucka et al. using *Artemisia abrotanum*. In this experiment, the herb is used to reduce the precursor for MgO NPs. The obtained MgO NPs, sized about 10 nm, showed increased antioxidant activity [119].

### 4.4. Iron Oxide Nanoparticles: Main Properties and Advantages

Another category of metal oxide NPs widely studied in biotechnology, medicine, and tissue engineering is iron oxide (FeO) NPs [120,121]. In this direction, maghemite (γ-Fe_2_O_3_), hematite (α-Fe_2_O_3_), and magnetite (Fe_3_O_4_) phases are the most studied iron oxide phases, providing remarkable physicochemical properties such as superparamagnetic, non-toxicity, environmental compatibility, and cost-effectiveness [122]. Moreover, FeO NPs have a higher surface area compared to iron ions and provide increased antimicrobial activity. Further, related to their antimicrobial activity, FeO NPs interact with bacterial cells through electrostatic interactions and the subsequent cell adhesion to the cell envelope. Through cell wall penetration, the NPs interact with proteins and lipids on the cell membrane and change the osmotic pressure, which will cause membrane disruption. Once reached in the cell membrane, the NPs can trigger oxidative stress and ROS generation, causing the disruption of DNA replication and inducing DNA double-strand breaks [123].

Related to the synthesis routes of FeO NPs, these could be obtained through physical and chemical synthesis routes. However, green synthesis routes have emerged as a solution to avoid using toxic solvents. As reducing and capping agents, fungi, plants, bacteria, polysaccharides, seaweed, and algae represent the solution in this regard. Among the mentioned naturally existing substances, plant and plant-based extracts are the most commonly selected sources due to their capacity to reduce precursors and their ability to stabilize NPs by using the existing biomolecules. The rich content of alkanes, phenols, aldehydes, and other functional groups in the extracts of leaves, seeds, flowers, and pericarp can reduce ion precursors and prevent FeO NP aggregation [124].

Hence, Yoonus et al. have demonstrated the successful green synthesis of α-Fe_2_O_3_ NPs by using Piper betel leaf extract, which provided superior antibacterial activity against *P. aeruginosa* and *Streptococcus mutans* and anticancer activity for A549 (lung cancer) cells [125]. Another study presented by Kirdat et al. has demonstrated the synthesis of FeO NPs ginger (*Z. officinale*) extract and has proven an increased antibacterial activity in the *E. coli* strain [126]. In addition, Table 5 also presents other studies about the green synthesis of metal and metal oxide NPs and their biological effects.

## 5. Applications of Green Synthesized Metal Oxide Nanoparticles in Tissue Engineering

**Figure 9:**
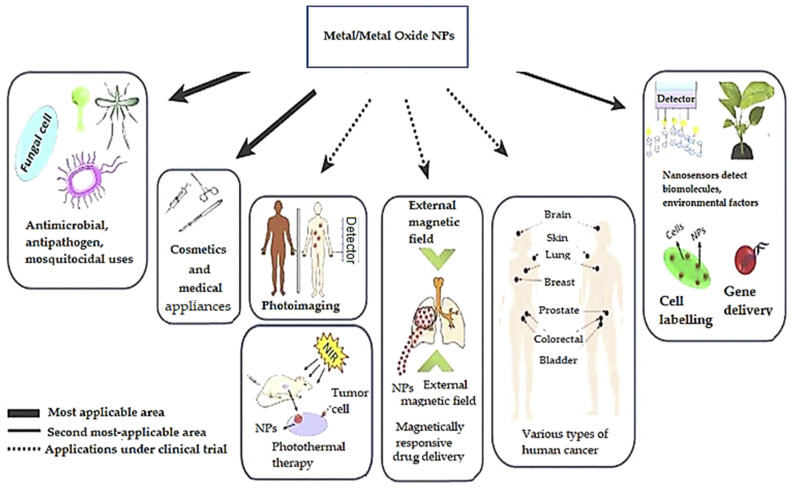
The main application of green synthesized metal and metal oxide NPs [136].

The latest advances in nanotechnology have brought noticeable benefits to tissue engineering. The synthesized biomaterials have been developed and optimized to be further applied in repairing or reconstructing damaged tissues/organs and designing intelligent drug delivery systems. With numerous applications of nanomaterials in tissue engineering, it is essential to select the appropriate nanomaterials for different tissue engineering applications. As mentioned, green synthesis comes as a solution to surpass all the side effects of conventional synthesis methods and provide an enhanced biological effect. The biogenic synthesis of metal and metal oxide NPs, the optimization process, and their further application in tissue engineering are still in progress. The following section will further discuss the most researched applications, as shown in Figure 9 and Table 6, of metal and metal oxide NPs.

### 5.1. Use of Green Synthetized Metal/Metal Oxide Nanoparticles for Scaffolds

One of the most researched applications of nanoparticles in tissue engineering is represented by their incorporation into scaffolds. As an effective approach to restoring damaged tissue/organs, scaffolds represent the safest route of tissue engineering by providing the proper structure for regeneration, supplemented with necessary bioactive substances [146]. The porous structure of scaffolds provides the necessary support for cell proliferation, attachment, migration, and differentiation [147,148]. Furthermore, the scaffolds should also offer the requested space for new tissue formation, vascularization, remodeling, and the necessary stability and mechanical strength. In addition, the functionality of the scaffolds could also be modified or even enhanced by introducing bioactive molecules or even nanoparticles [149].

The addition of functionalized nanoparticles and cells to a porous sponge represents a novel route to develop a tissue engineering scaffold further. These NPs can act as nanocarriers to gradually release the bioactive substance at the target site for long-term efficacy. Further, NPs can influence cell function and morphology transformations based on the types of biomaterials used. Sporadically, NPs can elicit inflammatory responses between cellular interactions. Still, they are easily integrated into implant design to adapt to inflammatory responses. Hence, multifunctional platforms have been developed by incorporating NPs into scaffolds. The pore size governs this incorporation, thus enhancing the resulting efficacy of the developed scaffolds. Therefore, it could be attested that integrating various types of NPs into advanced biomaterials is of great interest to many researchers [150]. Additionally, it is well known that metal and metal oxide NPs have native antibacterial activity. Their large surface area to volume ratio can provide an enhanced area of interaction, therefore increasing their antibacterial activity [151].

Thus, Vijayakumar et al. explained the efficient use of Ag, Au, Cu, and ZnO NPs and their incorporation in hydrogels with the aim of wound infection prevention. The obtained materials can absorb wound exudates and enhance keratinocyte migration, which leads to re-epithelialization and, at the same time, inhibits bacterial growth [152]. Furthermore, Li and Zhuang [153] related the use of MgO NPs, Ag NPs, and ZnO NPs to improve the antibacterial properties and stability of the chitosan-based nanocomposite. Both researchers have reported that bacterial growth has been effectively inhibited by the nanocomposite films. In another study, the group led by Mani [138] also obtained electrospun polyurethane scaffolds combined with green synthesized nickel oxide nanoparticles (NiO NPs) and groundnut oil. The developed nanocomposites have displayed improved physicochemical properties and enhanced mineral deposition in bone tissues. The scaffolds could be an ideal candidate for bone tissue engineering.

### 5.2. Green Synthetized Metal/Metal Oxide Nanoparticles for Drug Delivery

Considering drug delivery applications, nanotechnology can surpass the limitations given by conventional delivery, such as biodistribution and intracellular trafficking (molecular transport to specific organs, cell-specific targeting). Thus, NPs have the potential to enhance the stability and solubility of encapsulated molecules, promote membrane transport, and extend circulation times to increase efficacy and safety [154]. Early studies about NP formulations could not overcome the biological barriers necessary to provide successful delivery. However, numerous novel NP designs have been used and researched to provide controlled synthesis strategies, introduce bio-responsive moieties and complex architectures, and develop further targeting agents to improve delivery. Therefore, these NPs can be used as more complex systems (including in nanocarrier-mediated combination therapies) to modify multiple pathways, maximize therapeutic efficacy against targeted macromolecules, target specific phases of the cell cycle, or combat the mechanisms of drug resistance [155,156,157]. Lately, green synthesis of NPs has become an aid in the drug delivery process. Hence, diverse extracts are based on plants. Fungi and algae could represent a promising medium. The mentioned extracts are non-volatile, non-toxic, and non-hazardous media. Furthermore, the biological extracts offer an active biomolecule mixture (e.g., phenols, proteins, flavonoids) that provides increased stability in NP formation. For example, the NPs obtained using plants, polysaccharides, and polymers could have an enhanced biological effect [158]. Related to the green synthesis of metal and metal oxide NPs, it was concluded that the biomass source can provide pharmacological effects such as anti-diabetic, antibacterial, and anticancer activity. Furthermore, plant extract can also act as a potential compound that could aid the variation of the material during drug development [36].

Hence, Singh et al. [145] explained that ZnO NPs can act as efficient carriers for the targeted delivery of many anticancer drugs into malignant tumor cells. ZnO NPs show cancer cell-targeted toxicity through pH-dependent dissolution (low pH) in Zn^2+^ ions, which generate ROS species and induce cytotoxicity in cancerous cells. Lately, Zeghoud et al. [140] have explained the photosynthesis of ZnO NPs using *Mangifera indica* leaf extract. The developed composite functioned as an applicable anticancer drug with a cytotoxic effect comparable to cyclophosphamide at low doses against lung cancer (A549). The efficacy of green synthesized ZnO NPs as an anticancer drug was also conditioned to be dose-dependent, indicating that the anticancer activity of ZnO NPs peaked when higher doses were administered. When drugs, including curcumin, baicalin, paclitaxel, and doxorubicin, are placed on ZnO NPs as delivery vehicles, they demonstrate improved solubility and increased toxicity. In addition, Khan et al. [159] also explained that the surface of Au green synthesized NPs can be covered with the biomolecules provided by the plant/algae/fungi extract. Thus, the biosynthesized NPs can be functionalized or absorbed by plants, and peptide extracts could be applied as natural bio-linkers for targeted drug delivery to a specific tissue or cell type. In addition, the biocompatible nature of green-synthesized Au NPs with normal and abnormal cells makes them more suitable as a vector for drug delivery.

### 5.3. Green Synthetized Metal/Metal Oxide Nanoparticles for Bioimaging

Bioimaging is known as another biomedical application and advanced non-invasive technology applied to visualize the physiological processes and internal structures in living cells/organisms in a real-time manner. This is an effective and safe technique for monitoring biological functions without affecting usual life activities (breathing or movement). Further, bioimaging helps obtain data about the 3D nanostructure of the sample and explore tissues at the subcellular and multicellular scale. In recent years, nanomaterials have been considered ideal materials for nanoprobes as they can be accurately characterized using gel permeation chromatography or nuclear magnetic resonance and are effortlessly secreted by the body. Even though the studies of these NPs are limited, researchers are continuously looking for the development of new materials. The most well-known image-guided therapies are X-ray CT, fluorescence, magnetic resonance, and many others [160].

Each imaging technique has its advantages and disadvantages. Between multiple imaging modalities, nanomaterials, especially NPs, can be functionalized to be detectable, yielding synergistic advantages. Nanomaterials are more suitable for multimodal imaging because they are associated with smaller molecules. This process is caused by the larger surface area of NPs, which provides larger sites for functionalization and assists in designing them for multimodal sensing. In this direction, Kalpana et al. mentioned that Kalpana et al. explained how Gd-doped ZnO quantum dots (with sizes smaller than 6 nm) can be further employed for optical and magnetic resonance imaging. Further, the same group has also related that Singh et al. [39] explained using Fe_3_O_4_-ZnO magnetic quantum dots for cancer therapy and imaging.

Recently, Shete et al. [161] investigated the optical and biocompatibility properties of Co-Au nanocomposites containing Au nanocubes and Co nanorods. Au nanocubes developed near the nanorod surface or at the end of cobalt oxide NPs. The cobalt oxide NPs have been linked to anti-epidermal growth factor antibodies. The developed nanocomposite has been used to detect cancer cells in vitro in recent work. Further, the same group has also reported using cobalt oxide quantum dots and their detection in the cytoplasm when used in in vitro cell imaging. The used quantum dots have exhibited constant brightness under UV light without providing significant cytotoxicity.

## 6. Toxicity and Biocompatibility of Meta/Metal Oxide Nanoparticles

### 6.1. Overview of Toxicity and Biocompatibility Concerns with Metal/Metal Oxide Nanoparticles

Recent studies have strongly confirmed that metal and metal oxide NPS powerfully associate their physicochemical properties with the produced cytotoxicity. Thus, based on the shape, size, surface charge, dose, surface area, and solubility of NPs, the cytotoxicity can further be increased, decreased, or missing. The size represents one of the most important characteristics of NPs, as cellular update is the key criterion that causes cell death. Over the years, it has been determined that as the size of the NPs decreases, an enhanced cellular uptake will be produced [162,163,164]. Additionally, many studies have also concluded that small-sized NPs < 50 nm can enhance DNA damage and oxidation potential [165].

In addition, NP concentration or dosage is another parameter that is directly related to cytotoxicity. The study conducted by Ogholbeyg et al. [166] examined the concentration-dependent cytotoxicity of iron oxide nanoparticles using breast cancer cell lines. The results showed no toxicity up to a concentration of 200 μg/mL, while cancer cell death was shown to be dose-dependent. Moreover, the concentration of the reducing agent generally accelerates particle growth. However, the increased biological extract concentration leads to larger NP formation. In this regard, the increased NP’s size can be explained by a secondary reduction process that takes place on the metal nuclei due to the excess of reducing phytochemicals. Further, a small reductant concentration cannot provide sufficient biomolecules to start ion conversion. Even if the concentration of the biomolecules is reduced, this one could not be enough to start the reduction step as they would be quickly depleted by the metallic ions. Hence, an insufficient amount of biochemicals would be involved in the capping process, and therefore, the aggregation and precipitation of the NPs could occur. However, an excessive concentration of biomolecules could impair the nucleation of the NPs. The reducing ability of the biomass source is connected to its composition and biomolecule concentration [167].

The aggregation of NPs is another critical characteristic in determining their cytotoxicity, as larger particles could be cleared by macrophage cells in contrast to smaller particles, resulting in lower cytotoxicity of aggregated NPs larger than 100–200 nm. Moreover, a high concentration of NPs could increase agglomeration and decrease cytotoxicity compared to lower doses. Most agglomerations are larger than 100 nm, the threshold size for numerous side effects of micro/nanoparticles [80]. The shape of the NPs is another feature that deeply influences cytotoxicity behavior. The nanoparticles’ different shapes (circular, tubular, triangular, irregular, stellate, and many others) can influence their capacity to undergo phagocytosis and endocytosis. Related to the circular-shaped ones, it was concluded that they tended to be rapidly endocytosed [168]. The NPS surface charge, or functionalization, represents another factor that induces cytotoxicity. In this regard, the charge of NPs can impact their cellular uptake. Cells’ uptake of positively charged particles increases quicker than negatively charged ones. Hence, the enhanced cellular toxicity of positively charged NPs is connected with their increased cellular uptake. Nevertheless, the surface charge of nanoparticles can also control the agglomeration, leading to decreased or increased toxicity. Therefore, surface functionalization or coating can overcome this problem. Lately, Vales et al. have researched the effect of surface functionalization of Au NPs on cytotoxicity. The group compared 3 different surface charges of functionalized NPs using carboxylate (negative charge), ammonium (positive charge), and poly(ethylene glycol) (uncharged) for their cytotoxic effects. They have determined that positively charged NPs showed the highest cytotoxicity compared to others [169].

Finally, NP dissolution/solubility may correlate with the cytotoxicity of the particles. It is well known that metal and metal oxide NPs dissolve in the acidic environment of cells, amplifying toxicity. The phenomenon is referred to as a Trojan horse uptake mechanism, as NPs pass through the plasma barrier and release toxic ions to cause cell apoptosis or necrosis [165].

Green synthesized metal or metal oxide NPs are environmentally friendly, economical, and simple to produce on a large scale. Nevertheless, their cytotoxicity, compared to chemically synthesized NPs, has not been intensively discussed. Gowda et al. [165] have therefore compared the toxicity of the chemically and green synthesized Ag NPs in human umbilical vein endothelial and CHO cells. Their study has related the missing toxicity for green synthesized NPs and minor toxicity on rat cardiomyoblast cells. For the chemically synthesized NPs, toxicity has been observed even at small concentrations. In the same manner, other studies, such as the one reported by Kummara et al. [170], explained the toxic nature of chemically synthesized Ag NPs on human dermal fibroblast cells at 120–240 ppm. They have concluded that no toxicity was observed for green synthesized Ag NPs at 0–240 ppm in normal cells. However, green synthesized Ag NPs significantly inhibited the proliferation of NCI-H460 lung cancer cells at 160, 200, and 240 ppm concentrations. Chemically synthesized Ag NPs did not exhibit significant inhibition of proliferation in pulmonary cancer cells at similar concentrations. The authors also performed toxicity studies on brine shrimp. Green-mediated Ag NPs showed 100% and 56% mortality at 240 and 120 ppm concentrations. On the other hand, chemically obtained Ag NPs indicated 100% mortality at both 240 and 120 ppm concentrations in brine shrimp. All these findings suggest the exceptional biocompatibility of green-mediated metal/metal oxide NPs compared to their chemically synthesized counterparts.

In addition, Firdhouse et al. [171] have also reported the enhanced properties of biosynthesized nanomaterials over traditional synthetic precipitation NPs. Plant-mediated synthesized metal NPs have increased biocompatibility over chemical, physical, and microbe-assisted synthesized NPs.

### 6.2. Results of Toxicity and Biocompatibility Studies for Green Synthetized Metal/Metal Oxide Nanoparticles

A growing interest in using nanoparticles in biomedical engineering has raised many concerns about their biocompatibility and cytotoxicity. Nanomaterials behave differently than bulk materials due to the surface-to-volume ratio. These parameters also directly impact the chemical reactivity of the materials and their toxicity [172]. Techniques for assessing the biocompatibility of nanostructured materials are not completely comprehensive, as it is a constantly growing domain where biomaterials are constantly being developed for tissue engineering and regenerative medicine applications. The biomaterial’s cytocompatibility also impacts the output of in vitro experiments. Determining parameters such as nanoparticle concentration and cell type requires a detailed understanding of estimated nanoparticle exposure and its metabolic activity in the human body. Therefore, selecting appropriate methods for in vitro and in vivo toxicity analysis will provide adequate insight into the mechanism of toxicity of nanomaterials. It can be effectively used in regenerative medicine. This section will further provide some insight into their biocompatibility and toxicity assessment [173].

**Figure 10:**
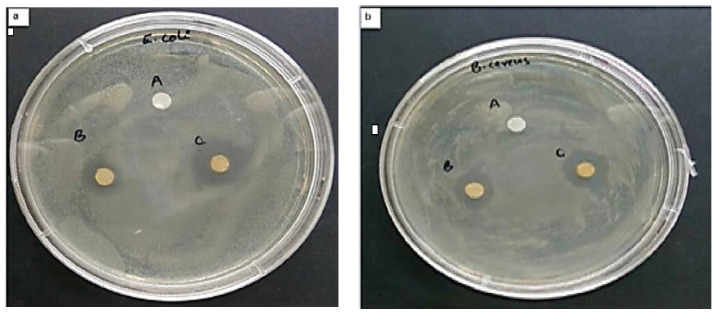
HNTs (A), HNTs/cur (B), and HNTs/cur/AgNPs (C) antibacterial activity against *E. coli* (**a**) and *B. cereus* (**b**) [174].

In Figure 10, Rahman et al. [174] have explained the antibacterial properties of HNTs/cur/Ag NPS. The synthesis of highly biocompatible Ag NPs intercalated in the halloysite nanoclay (HNT) using curcumin (cur) as a reducing agent. The green synthesis of Ag NPs has been performed first by the complexation or intercalation between curcumin and HNT. For the next step, the in-situ reduction of silver nitrate to form Ag NPs in HNT has been performed. Curcumin has been used as a strong reducing agent for silver nitrate. Figure 10 displays the antibacterial activity of A—HNTs, B—HNTs/cur, and C—HNTs/cur/AgNPs against *B. cereus* and *E. coli.* Against both strains, it can be seen that pure HNTs have no inhibition capacity. The HNT/cur capacity can be observed with a radial inhibition zone of 13 mm for *B. cereus* and 8 mm for *E. coli*. The radial zone of inhibition of HNT/cur/AgNP is larger than that of HNT/cur for both *B. cereus* (16 mm) and *E. coli* (17 mm). The samples without Ag NPs have, therefore, provided a smaller antibacterial effect. The biosynthesized nanocomposite has exhibited excellent antibacterial activity, and it can thus be applied as an ideal material for biomedical applications.

**Figure 11:**
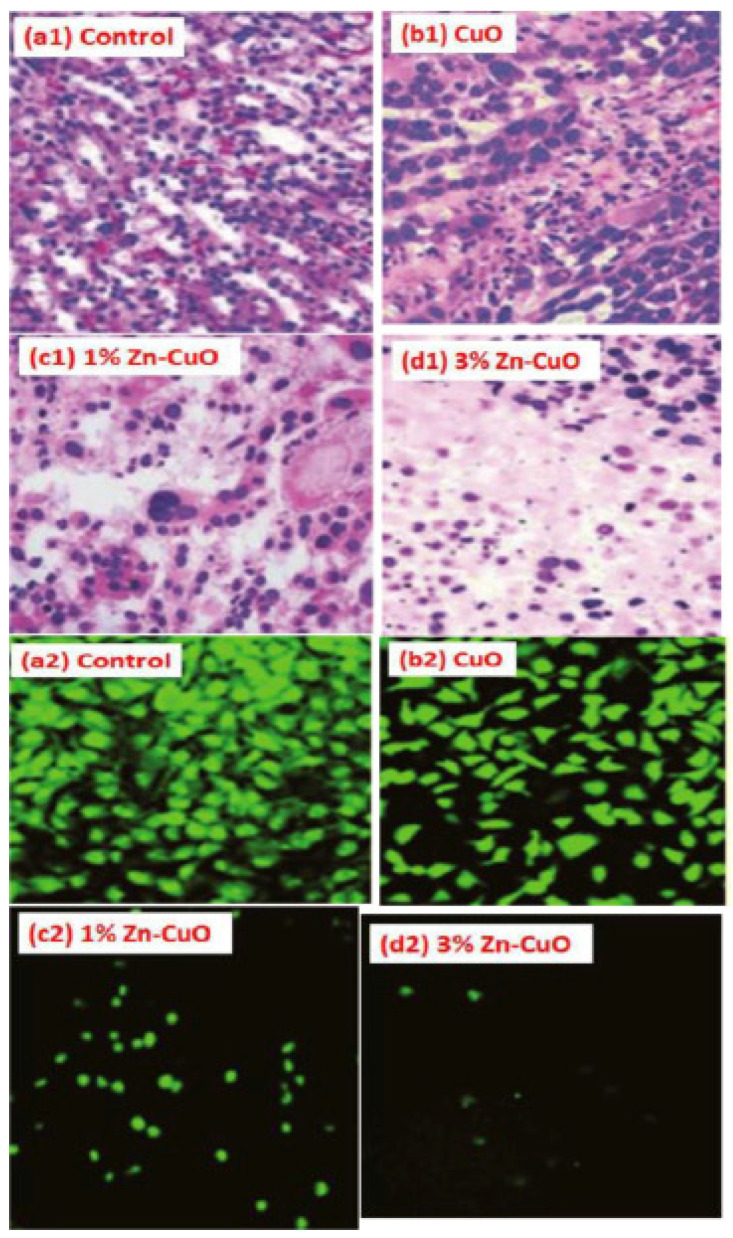
Hematoxylin-eosin-stained histological images of kidney tumor (A-498) cells (**a1**–**d1**) after treatment with NPs in different concentrations and CLSM images (**a2**–**d2**) of kidney tumor (A-498) cells treated with Zn-CuO NPS by FITC staining, emitting green fluorescence (scale bar: 30 μm) [174].

The same group has also reported the use of *Duchesnea indica* for the development of Zn-doped CuO NPs (Zn/CuO NPs) exhibiting a spherical morphology. The green synthesized Zn/CuO NP’s anticancer ability was widely studied using A-498 cancer cells and normal human epithelial cells at different concentrations (10, 20, 40, 80, and 160 μg/mL). Figure 11 presents the microscopic images of dead cells on tumor cells treated with green-synthesized Zn/CuO NPs with (hematoxylin-eosin) H&E and (Fluorescein isothiocyanate) FITC staining. For histological and confocal laser scanning microscopy images (CLSM), the maximum number of cells disappeared when treated with 3% Zn/CuO NPs. Compared with each other, improved antitumor activity has been observed in Zn-doped NPs than in undoped materials. Further, the antitumor activity of 3% Zn/CuO NPs mostly depends on their polar surface area, size, and morphology, as well as the concentration of Zn ions doped on the CuO surfaces [174].

**Figure 12:**
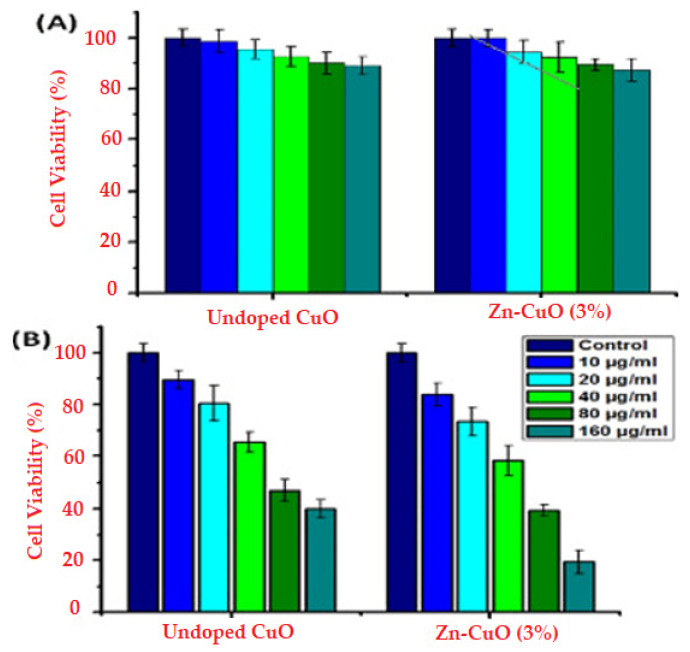
The CuO and Zn-doped CuO NPs cytotoxic activity on (**A**) renal proximal epithelial (HK-2) cells and (**B**) on A-498 kidney cancer cells [174].

Figure 12 presents the cell viability assessment of green-synthetized Zn-doped CuO NPs. It was concluded that there are no significant changes in normal renal epithelial cell viability, with an 85% viable cell rate at higher sample concentrations. Figure 12A proves that the synthesized NPs gave an outstanding inhibition rate of treated cells on 498 kidney tumor cells. Therefore, the assessment of the antitumor activity has shown that the synthesized Zn/CuO NPs gave incredible cancer cell inhibition against the proliferation of A-498 cells. To conclude, the results of the antitumor analysis of Zn/CuO NPs with 3% Zn ions established that an 80% inhibition rate means their maximum capacity (Figure 12B) [174].

Furthermore, the study led by Divakaran et al. [175] has explained the Au NPs biosynthesis and successful growth inhibition of breast cancer cells (MCF-7) without inducing toxicity for (epithelial, human breast) MDA-MB-231 cells. Similarly, Oh et al. [176] also developed Au NPs using a *C. sinensis* fruit extract. The size of the Au NPs was in the range of 20–40 nm and has been further tested for cytotoxicity against breast cancer cells. In this regard, cytotoxicity was evaluated against the MCF7 cell line, providing significant cell growth inhibition. Recently, Nandhini et al. [116] have fabricated a novel antimicrobial composite based on green synthesized ZnO NPs (using *Ilex paraguariensis* leaf extract), incorporated in electrospun polyallylamine hydrochloride (PAH) and polyacrylic acid (PAA) fibers for wound healing applications. ZnO NPs demonstrated high antimicrobial activity against various microorganisms involving drug-resistant bacteria, being less cytotoxic against L929 mouse fibroblast cells. Moreover, the ZnO NP’s antimicrobial activity against gram-positive bacteria (*S. aureus*) was higher than that against gram-negative bacteria (*E. coli*). Thus, the minimum inhibitory concentration (MIC) for *S. aureus* was 35 g mL^−1^, while *E. coli* showed higher resistance, even at a concentration of 100 g mL^−1^ with 70% viability.

**Figure 13:**
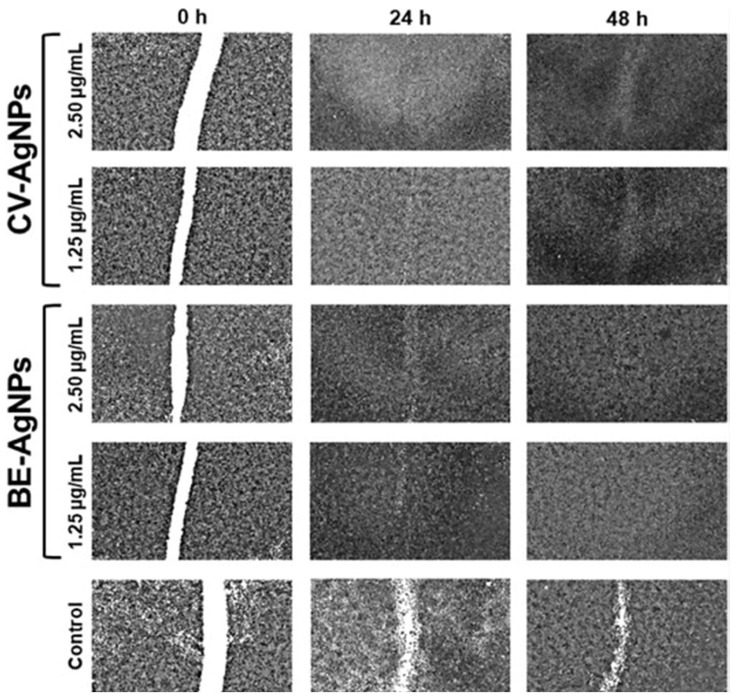
In vitro wound healing ability of BE-AgNPs and CV-AgNPs at 2.50 g/mL and 1.25 g/mL in the L929 cell line during 24 and 48 h [116].

Furthermore, the same group has explained Ag NPs’ synthesis using *Boletus edulis* (BE-AgNPs) and *Coriolus versicolor* (CV-AgNPs) through microwave-assisted green synthesis. The average particle size of BE-Ag NPs varies by ~87.7 ± 0.8 nm, and that of CV-AG NPS by ~86.0 ± 3.8 nm. Moreover, the NPs exhibit negative zeta potential on their surfaces, which enhances their stability. These NPs show promising antimicrobial activity against gram-negative bacterial strains (*Klebsiella pneumoniae* and *Pseudomonas aeruginosa*) and gram-positive bacterial strains (*Enterococcus faecalis* and *Staphylococcus aureus*). Although both types of NPs presented significant inhibitory effects on *Candida utilis* fungal strains, they proved ineffective against *Candida albicans*. BE-AgNPs and CV-AgNPs proved anti-proliferative activity in HUH-7, MCF-7, and HT-29 cancer cell lines in a dose- and time-dependent manner. From the results of the MTT cell proliferation assay, BE-AgNPs were found to provide a higher anti-proliferative activity than CV-AgNPs in all three cell lines at 48 h. CV-AgNPs and BE-AgNPs could induce the migration of L929 cell lines (murine fibroblast cells) and successfully heal wounds at low concentrations. When L292 cells were treated with 2.50 μg/mL and 1.25 μg/mL of BE-AgNPs and CV-AgNPs, the migration of fibroblast cells was higher than the control at 24 and 48 h. Figure 13 presents the wound healing capacity of BE-Ag NPs and CV-AG NPS by enhancing L929 murine fibroblast cell migration in a dose- and time-dependent assessment. First, the viability profile of L929 cells after exposure to various doses of BE-AgNPs and CV-AgNPs was examined by MTT cell proliferation analysis. Fibroblast cells cured with 2.50 μg/mL and 1.25 μg/mL of BE-AgNPs and CV-AgNPs migrated significantly compared to control cells after 24 h and 48 h. Healthy cell migration from the wound border is a substantial biological process in treating chronic and acute wounds. Further, promoting cell migration to the wound area accelerates the replacement of inflammatory cells with collagen and healthy tissue. The experiments conducted proved in vitro fibroblast migration and the fact that the NPs can be successfully applied in wound healing applications [116].

**Figure 14:**
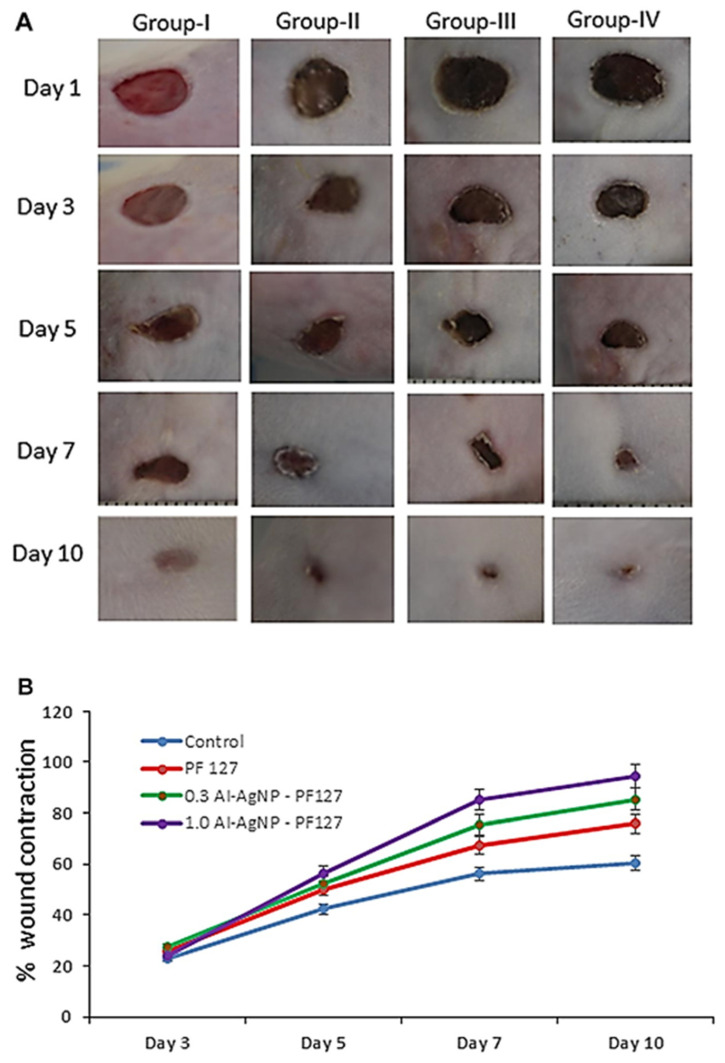
Wound healing process in vivo mice studies: (**A**) Wound healing effect on control (group-I), pristine PF127 hydrogel (group-II), 0.3 mg AI-AgNPs-PF127hydrogel (group-III), and 1.0 mg AI-AgNPs-PF127 hydrogel (group-IV), and (**B**) wound contraction percentage with time [177].

In addition, the group led by Chinnasamy has managed to synthesize Ag NPs by using *Azadirachta indica* leaves (AI-AgNPs), obtaining, therefore, spherical nanoparticles with a ~30 nm diameter. For the next step, they developed a pluronic F-127 (PF127)-based hydrogel, which was further functionalized with AI-AgNPs. The obtained hydrogels exhibited significant antimicrobial activity without causing undesirable effects such as redness and skin dryness. Hence, Figure 14A,B presents the biocompatibility and healing abilities of the hydrogels. For the pure PF127 hydrogel, 0.3 and 1 mg AI-AgNPs-PF127 achieved wound contraction rates around 75.77, 85.52, and 94.54 on the 10th day, respectively. Moreover, AI-AgNPs-PF127 hydrogel improved the wound contraction rate when topically applied to mice. The wound healing quantitative analysis implied measuring the initial wound size (1st day) and the healing process towards wound closure (10th day). For the control group, the healing rate, in terms of percentage wound contraction, was 23.12 on the 3rd day, 42.33 on the 5th day, 56.11 on the 7th day, and 60.42 on the 10th day. Further, the mice healed with pure PF127 hydrogel exhibited an improved wound contraction (compared with the control group) of 25.46 on the 3rd day, 50.11 on the 5th day, 67.54 on the 7th day, and 75.77 on the 10th day. The group treated with 0.3 mg AI-AgNPs-PF127 hydrogel displayed higher wound contraction rates, such as 27.22 on the 3rd day, 52.32 on the 5th day, 75.44 on the 7th day, and 85.44 mg on the 10th day. Lastly, the group treated with 1.0 mg AI-AgNPs-PF127 hydrogel reached the highest wound contraction rate: 24.25 on the 3rd day, 56.43 on the 5th day, 85.23 on the 7th day, and 94.54 on the 10th day, as shown in Figure 14A [177].

In conclusion, the in vivo wound healing assessment of functionalized scaffolds showed almost complete wound closure by using 1.0 mg AI-AgNPs-PF127 hydrogel on day 10, thus confirming their healing potential (Figure 14B). The 1 mg of the AI-AgNPs-PF127 hydrogel-cured group had significantly faster healing activity compared to the control group [177].

Hence, we can summarize that a thorough assessment of biocompatibility and cytotoxicity is extremely important for the green synthesized nanoparticles, specifically for the ones applied in biomedical applications. The studies are still limited at this moment, and further research must be performed in this regard.

## 7. Conclusions and Future Perspectives

In recent years, nanotechnology has gained great attention among researchers, especially in the materials science field. Considering this, in this review, we have discussed the basic properties, preparation, and use of different types of nanoparticles and their outstanding application in tissue engineering. Their use in tissue engineering applications is essential for the repair or regeneration of damaged tissues. Regarding existing nanotechnology, more and more researchers are trying to develop novel biomaterials using diverse combinations of numerous nanomaterials. Furthermore, it was concluded that even if NPs can be obtained via many chemical and physical synthesis routes, they are also characterized by their high prices, the need for and use of high temperatures and pressure, and their negative impact on the environment (potential generation of hazards or exertion of carcinogenicity/environmental toxicity). Hence, increased attention is now given to the greener synthesis pathways using diverse biomass sources (plants, fungi, algae, or bacteria). Green synthesis is also well known for its quick, simple, eco-friendly, inexpensive, and safe fabrication of nanoparticles. Another important advantage of green synthesis is the use of non-toxic solvents and efficient production processes.

In addition, the green synthesized metal or metal oxide NPs can be successfully applied for diverse biomedical applications such as tissue engineering, drug delivery, cancer therapy, and even bioimaging. The use of plants or plant extracts has provided a synergetic effect, which is beneficial as it can produce less toxicity in the surrounding medium. The shape, size, and reaction rate of the developed NPs are strongly influenced by various experimental parameters such as reactant concentration, reaction time, pH, temperature, salt concentration, and many other parameters. The parameters could also influence the polydispersity of the developed materials, and therefore, they still require continuous research. Further, most of these green synthesis strategies are still in the research stage, and their challenges must still be solved. These encompass nanoparticle stability and aggregation, managing crystal growth, morphology, and size.

Additionally, the separation and purification of NPs is another vital parameter that needs to be further explored. Metal NPs produced by plants and/or plant extracts are more stable compared to those formed by different organisms. In this regard, plants (especially plant extracts) can reduce metal ions faster than fungi or bacteria. Furthermore, to use an easy and safe ecological method in the scale-up and industrial production of well-dispersed metal NPs, plant extracts are the ideal components of plant biomass or living plants.

Most research studies use solvent or extract compositions without adequate quantification of each component to create an unreproducible synthetic pathway that is difficult to scale. Although numerous studies concluded that metal NPs exhibited higher toxicity, recent research demonstrated that the proper dosage, size, and distribution make these materials less toxic. The main limitation of biosynthesis is the lack of proper optimization of influencing parameters and the selection of the ideal solvent. The scope of this optimization is to provide a comparable assessment related to the toxicity and biocompatibility of the NPs. The nanoparticles’ biostability depends on their shape and surface charge, features influenced by enzymes, phenols, and vitamins involved in the production medium.

Furthermore, it can also be concluded that most of the latest studies follow the synthesis method, physicochemical characterization, and in vitro evaluation by using different cell lines (depending on their application) or microbes. The number of research studies that have proven the wound healing ability and use as topical applications of biosynthesized NPs is still limited. The percentage of reports that provide in vivo particle biodistribution and kinetic profiles is minimal, even if this is a principal criterion for regulatory approval for most nanomedicines. In addition, degradation pathways require special attention, as their simple in vitro degradation assessment is unsatisfactory. Therefore, the environmentally friendly synthesis of nanoparticles and their biomedical applications still require special attention in future studies.

## Figures and Tables

**Table 1:** Mechanism of synthesis using different biomass sources for green synthesis of metal and metal oxide NPs [33].

Sources	Potential Modifications in Nanomaterial Synthesis	Advantages	Disadvantages
Plant	Secondary metabolites (Saponins, Tannins, alkaloids, and other metabolite compounds) act as capping and stabilizing agents	Low cost, eco-friendly, easily scaled up, no need for high pressure, temperature, or energy, lack of toxic precursors, no need for culture maintenance, biocompatible	Harmful effects are not fully known; plants generate low yields of secreted proteins, which can decrease the synthesis rate
Fungi	Involve the intracellularly or extracellularly reducing enzyme and biomimetic mineralization	Large-scale NP fabrication, inexpensive, energy-efficient, increased metal accumulation, superior wall-binding capacity, eco-friendly, easy biomass handling	Low reproducibility, difficult genetic manipulation, pure NPs cannot be obtained without using other solvents, broad particle size distribution
Algae	Polysaccharides function as capping and stabilizing agents for NP synthesis	Lack of toxic byproducts, eco-friendly, biocompatible, able to grow under diverse conditions, low cost, easy to handle, do not require cellular maintenance	Time-consuming preparation of algae culture, limited scale-up fabrication, reproducibility method needs to be improved, limited size control, not all species can be used in NPs synthesis
Bacteria	Bacteria can reduce metal ions into metal by nitrate-dependent reductase or NADPH-dependent reductase enzyme	Nontoxic, biocompatible, ecofriendly, does not demand too much energy, inexpensive	Isolation, microbial sampling, storage, and culturing needed, time-consuming procedures, hard to control the morphology of the NPs

**Table 2:** Advantages and drawbacks of green synthesis [34].

Advantages	Drawbacks
(a) Low cost; (b) Facile implementation; (c) Eco-friendly approach, toxic solvents, and chemicals are excluded in this method; (d) No contamination compared to chemical and physical synthesis; (e) The ability to create well-defined NPs of a specific size and shape; (f) Reuse of waste materials; (g) It does not require high pressure and temperature; (h) Use easily accessible and renewable raw materials; (i) Effective in large-scale production of NPs.	(a) Nanotechnology has raised the stakes for human health; (b) Limited studies have been conducted on the bioaccumulation and toxicity of NPs in the environment; (c) Due to their small size, NPs can easily enter the body and cause inhalation problems or several other fatal diseases; (d) Compared to chemically generated NPs, green synthesis is not yet widely used at the industrial level.

**Table 3:** The main advantages and disadvantages of green synthesis solvents.

Solvent	Advantages	Drawbacks
Water	Low cost, natural source, obtained with ease, high availability, aids in the recovery of inorganics contained in biomass, avoids biomass drying necessity, and highest safety.	Not all reactions can occur in water; with lower solubility or most organic compounds, the solubility could be increased by using other co-solvents, surfactants, or pH control.
Ionic liquids	High thermal stability, recyclable, non-flammable, no measurable vapor pressure, immiscible with water or organic solvents.	Expensive compared with other organic solvents, they must be produced by using other solvents. An extraction step is necessary, as is the need for washing the obtained materials.
Supercritical Fluids	Good mass transfer, increased diffusion rate, non-toxicity; decreased solvation, facile control over properties, facile removal, recyclable, increased gas solubility, non-flammable.	Potential heat transfer issues, the necessity of high-pressure equipment, a relatively poor solvent that is relatively inert but reacts with powerful nucleophiles, and the difficulty of scaling up.

**Table 4:** Green synthesized noble metal NPs and their biological activity.

Type of Nanoparticles	Biomass Source	Biological Effect	Strains	Reference
Au NPs	cashew leaf extract	antimicrobial activity, antibacterial activity	*E. coli* and *B. subtilis*	[91]
	black and green tea leaves	antibacterial activity	*S. aureus* and *K. pneumoniae*	[92]
Ag NPs	*Boerhaavia diffusa*, and *Prunus persica*	antibacterial, antifungal, or even anticarcinogenic activity	*F. branchiophilum* and *E. coli*	[84]
	*Punica granatum* green leaves	anticancer activity	human liver cancer cells (HepG2)	[93]
Pt NPs	*Prunus yedoensis* tree gum extract	antifungal activity	*C. fulvum*, *C. acutatum*, *P. drechsleri*, *P. capsica*, and *D. bryoniae*	[90]
	* Syzygium aromaticum * extract	antibacterial activity	*S. aureus*, *S. mutans*, *E. coli*, *E. faecalis*	[94]

**Table 5:** Green synthesized metal/metal oxide NPs and their biological activity.

Type of NPs	Biomass Source	Biological Effect	Strains	Reference
CuO NPs	*Polyalthia longifolia* leaf extract	Antibacterial activity	*Staphylococcus aureus*, *E. coli*, *Pseudomonas aeruginosa*, and *Streptococcus pyogenes*	[127]
	tea leaves	Antifungal activity	*Fusarium solani*	[128]
	*Cissus quadrangularis*	Antifungal activity	*A. flavus* and *A. niger*	[128]
ZnO NPs	*Anabaena cylindrical algae* extract (algal extract)	antimicrobial activity	against *Pseudomonas aeruginosa* and *Staphylococcus aureus*	[111]
	fungal culture filtrate of *Alternaria tenuissima*	antimicrobial, anticancer, antioxidant	*Pseudomonas aeruginosa*, *Klebsiella pneumoniae*, *Staphylococcus aureus*, *Candida albicans*, *Alternaria solani*, *Aspergillus niger* and *Fusarium oxysporum*	[129]
MgO NPs	Piper Betle leaf extract	antibacterial activity	*B. subtilis* and *P. aeruginosa*	[130]
	*Costus pictus* D. Don leaf extract	antimicrobial and anticancer activity	*S. aureus*, *B. subtilis*, *E. coli*, *S. paratyphi*, *C. albicans* and *A. niger*	[131]
α-Fe_2_O_3_ NPs	*Laurus nobilis* L. leaves extract	antimicrobial activity	*Listeria monocytogenes*, *Aspergillus flavus* and *Penicillium spinulosum*	[132]
Fe_3_O_4_ NPs	*Bombax malabaricum* extract	antimicrobial activity	*Staphylococcus aureus*, *Bacillus halodurans*, *Micrococcus luteus*, *Escherichia coli*, *Aspergillus niger* and *Aspergillus flavus*	[133]
	*Nigella sativa* seeds extract	antibacterial and anticancer activity	*Salmonella typhi*, *Escherichia coli*, *I* A549 and HCT116 cell lines	[134]
	*Mikania mikrantha* leaf extract	antimicrobial activity	*Bacillus cereus*, *Acinetobacter johnsonii*, *Pseudomonas aeruginosa*, *Achromobacter spanius* and *Chromobacterium pseudoviolaceum strain*, *Aspergillus niger*, *Penicillium citirinum*, *Fusarium oxysporium*, and *Candida albicans*	[135]

**Table 6:** The main applications in tissue engineering of green synthetized metal and metal oxide NPs.

Type of NPs	Material	Application	Reference
Pt-loaded calcium phosphate (CaP)	Scaffolds	Bone tissue engineering	[88]
poly-L-lactic acid (PLA) nanofibers containing Au, Pt, and TiO_2_ NPS	Scaffolds	Bone tissue engineering	[137]
polyurethane/nickel oxide NPs (*Plectranthus amboinicu* leaf extract)/groundnut oil	Scaffolds	Bone tissue engineering	[138]
lignin/graphene oxide/ZnO(*Camellia sinensis* extract)/silk fibroin	Scaffolds	Biofilm modulation	[139]
ZnO NPs (*Mangifera indica* leaf extract)	Nanocarrier	Lung Cancer drug delivery	[140]
Poly-acrylamide-MgO NPs-acyclovir	Hydrogel	Vaginal drug delivery	[141]
MgO NPs (*Abrus precatorius* extract)	Nanocarrier	Cancer drug delivery	[142]
AgS NPs (*Pleurotus ostreatus* mycelium)	Nanoparticles	Bioimaging, biosensors	[143]
Ag NPs (*Olax scandens* leaf extract)	Nanoparticles	Bioimaging	[144]
ZnO NPs (*Gracilaria edulis* extract)	Nanocarrier	Cervical cancer drug delivery	[145]
ZnO NPs (*Aspergillus terreus*)	Nanobiocomposite	Breast cancer drug delivery	[145]

## Data Availability

Not applicable.
